# Exploring the Therapeutic Potential of Natural Compounds in Psoriasis and Their Inclusion in Nanotechnological Systems

**DOI:** 10.3390/antiox13080912

**Published:** 2024-07-28

**Authors:** Ana Flavia Burlec, Monica Hăncianu, Bianca Ivănescu, Irina Macovei, Andreia Corciovă

**Affiliations:** 1 Department of Drug Analysis, Faculty of Pharmacy, “Grigore T. Popa” University of Medicine and Pharmacy, 16 University Street, 700115 Iasi, Romania; ana-flavia.l.burlec@umfiasi.ro (A.F.B.); maria.corciova@umfiasi.ro (A.C.); 2 Department of Pharmacognosy, Faculty of Pharmacy, “Grigore T. Popa” University of Medicine and Pharmacy, 16 University Street, 700115 Iasi, Romania; monica.hancianu@umfiasi.ro; 3 Department of Pharmaceutical Botany, Faculty of Pharmacy, “Grigore T. Popa” University of Medicine and Pharmacy, 16 University Street, 700115 Iasi, Romania

**Keywords:** psoriasis, plant species, secondary metabolites, clinical studies, nanotechnology

## Abstract

Psoriasis is a chronic inflammatory disease that affects around 2–3% of the world’s population. The treatment for this autoimmune disease still remains centered around conventional methods using synthetic substances, even though more recent advancements focus on biological therapies. Given the numerous side effects of such treatments, current research involves plant extracts and constituents that could prove useful in treating psoriasis. The aim of this narrative review is to highlight the most known representatives belonging to classes of natural compounds such as polyphenols (e.g., astilbin, curcumin, hesperidin, luteolin, proanthocyanidins, and resveratrol), alkaloids (e.g., berberine, capsaicin, and colchicine), coumarins (psoralen and 8-methoxypsoralen), and terpenoids (e.g., celastrol, centelloids, and ursolic acid), along with plants used in traditional medicine that could present therapeutic potential in psoriasis. The paper also provides an overview of these compounds’ mechanisms of action and current inclusion in clinical studies, as well as an investigation into their potential incorporation in various nanotechnological systems, such as lipid-based nanocarriers or polymeric nanomaterials, that may optimize their efficacy during treatment.

## 1. Introduction

Medicinal plants have long been used worldwide for the treatment of various disorders. Different metabolites found in such plants proved to be effective against a large number of conditions and pathogens and, therefore, were considered for drug development, with roughly half of the medicines marketed nowadays being obtained from natural sources [1,2]. Natural compounds show great structural variety and many classes of active principles have been described so far, ranging from polyphenols to terpenoids and alkaloids [3]. For these substances, numerous biological activities have been demonstrated, including antioxidant, antimicrobial, anti-inflammatory, and anticancer properties, which illustrate their significant therapeutic potential in conditions such as cancer and inflammatory and auto-immune diseases [4,5,6].

Much attention has been drawn lately to the potential of natural compounds in preventing or even treating different types of cancer and cardiovascular and neurological diseases through several mechanisms [7,8,9]. However, the treatment of autoimmune diseases remains centered around conventional methods using synthetic substances, even though recent advancements focus on biological therapy. Therefore, more research could be carried out in order to identify plant metabolites that could present implications in treating such conditions, thus lowering the incidence of adverse reactions and enlarging current treatment options [10].

One of the most prevalent autoimmune diseases is considered to be psoriasis, a chronic inflammatory disease that affects around 2–3% of the world’s population [11,12]. For most patients, the onset of the disease is usually between 15 and 30 years of age, with a maximum incidence of around 22.5 years, even though it can appear at almost any age [13]. Around 85% of patients exhibit mild to moderate symptoms [14]. There are several types of psoriasis such as vulgaris (plaque), guttate, pustular, erythrodermic, and flexural (inverse), with plaque psoriasis being the most common type [15].

Psoriasis involves the formation of skin lesions, which can be either localized or generalized, commonly symmetrical, erythematous, well defined, and covered with white scales [16]. These lesions cause pruritus, a burning sensation, and pain. Moreover, in up to 69% of all patients with psoriasis, nail involvement (nail pitting) is present, which can lead to onycholysis in severe cases. In addition to these symptoms, psoriatic arthritis may also develop. Nonetheless, this condition has a large psychological impact and patients have to endure significant social stigma [14,16]. It is also noteworthy that patients with psoriasis are at higher risk of developing cardiovascular and gastrointestinal diseases, metabolic syndrome, and cancer [17].

Psoriasis is considered a painful, and sometimes disabling disease for which no cure has yet been found. The classic treatment includes three conventional therapeutic approaches: topical therapy (corticosteroids, vitamin D_3_ analogues, coal tar, dithranol, and keratolytics), phototherapy (narrowband and broadband ultraviolet B, psoralen plus ultraviolet A), and traditional systemic therapies (cyclosporine, acitretin, methotrexate, and fumaric acid esters) [18]. More recently, biologics such as etanercept, infliximab, adalimumab, certolizumab pegol, ustekinumab, and secukinumab have also been included in the therapeutic scheme for more severe cases of psoriasis and act by targeting T cells or by blocking proteins such as tumor necrosis factor-alpha (TNF-α) and interleukins (e.g., IL-1, IL-2, IL-6, IL-8, IL-10, IL-12, IL-17, IL-20, IL-22, IL-23, and IL-36), which are some of the most important cytokines responsible for psoriasis symptoms [19,20]. 

Given the fact that psoriasis is a chronic disease, a long-term treatment strategy must be considered, therefore the numerous contraindications and side effects of current therapies can sometimes prove difficult to handle for patients [21]. The limitations of such treatment options raise the need to discover new potentially effective agents with fewer inconveniences. Therefore, purified natural compounds could present a viable alternative for the treatment of psoriasis, given their numerous biological properties, including anti-inflammatory and antioxidant activities.

Among the main classes of natural products that might present beneficial roles in the treatment of psoriasis, polyphenols, flavonoids, alkaloids, coumarins, and terpenoids are to be mentioned [22,23,24]. Even though many plant species are considered useful for the treatment of this condition, it is usually purified compounds or mixtures containing such compounds that are more likely to find use in medicinal products [25].

Therefore, the aim of this review is to highlight the most known representatives belonging to such classes of natural compounds, along with plants used in traditional medicine that could present therapeutic potential in psoriasis, to offer a general overview of their implied mechanism of action and their inclusion in clinical studies, as well as to check their potential incorporation in different nanotechnological systems that could maximize their effectiveness during treatment.

This narrative review provides a novel perspective by combining recent findings on natural compounds that can be used for psoriasis treatment and data on their inclusion in nanosystems. Besides reviewing the therapeutic effects of plant extracts and substances, the paper also examines the usefulness of their integration in advanced nanotechnological systems, as well as recent clinical data, thus offering an updated perspective on potential psoriasis treatment with minimized side effects.

## 2. Pathogenesis of Psoriasis

Psoriasis is associated with epidermal hyperproliferation linked to the hyperactivation and abnormal or lack of differentiation of keratinocytes [26,27]. The etiology of this condition has not been entirely elucidated, but a genetic predisposition appears to be involved [28]. It is believed that the interaction between this genetic predisposition and certain triggering factors is what causes this condition to manifest, which makes psoriasis a multifactorial genetic disease [29]. Different studies have shown that there are multiple genetic loci linked to this disease, such as PSORS1-PSORS10, with PSORS1 being the most investigated susceptibility region [30]. Moreover, it has been demonstrated that HLA-Cw6 is the major PSORS1 risk allele involved in early-onset psoriasis [31]. Triggering factors generally include endocrine disfunction, infections with certain bacteria, viruses, and fungi, alcohol consumption, obesity, smoking, administration of specific medicines, psychological stress, skin injury, and prolonged exposure to sunlight [32,33].

Studies have shown that cytokines such as IL-23, IL-17, IL-1, and TNF-α play important roles in psoriasis pathogenesis and that molecules that target such substances exhibit significant results in the treatment of plaque and pustular psoriasis [28,34]. 

Several researchers have proven that the IL-23/IL-17 pathogenesis pathway is controlled by the skin and gut microbiota through its metabolites [35]. For some patients, a decrease in the population of *Lactobacillus* spp., *Burkholderis* spp., and *Propionibacterium acnes* has been noticed, when compared to healthy individuals [36]. An abnormal skin microbiome has been linked to psoriasis, involving specific modifications in the abundance of *Actinobacteria*, *Firmicutes,* and *Proteobacterium*, and increased amounts of *Staphyloccocus* and *Streptococcus* [37]. Moreover, a reduction in beneficial bacteria such as *Faecalibacterium prausnitzii* and *Akkermansia muciniphila*, and an increase in *Escherichia coli* in the gut microbiome have been reported in psoriasis [38,39]. This disease has also been linked to different types of fungi, such as *Malassezia* and *Candida* species, as well as to viruses (e.g., human papillomavirus) [40].

An important regulatory factor in inflammation, cell proliferation, and apoptosis, NF-kB is also believed to play a key role in the pathogenesis of psoriasis. Numerous cell types found within psoriatic lesions such as keratinocytes, dendritic cells, macrophages, and T lymphocytes, as well as chemokines and cytokines, are correlated with NF-kB signaling [41,42]. High amounts of NF-kB enable keratinocyte hyperproliferation and, therefore, many existing treatments for psoriasis also act by altering NF-kB signaling [41,43]. Moreover, the TNF-α and TH-17/IL-23 pathways are crucial in the pathogenesis of this condition, and, consequently, TNF-α inhibitors and IL-12/IL-23 and IL-17 inhibitors have been developed [30,41,44]. However, these medications present several disadvantages such as a considerable risk of infection and malignancy, the onset or aggravation of heart failure, as well as an increased risk of suicidal thoughts and behavior [30]. Therefore, new solutions for the treatment of psoriasis can be sought in the realm of natural products, with a focus on phyto-complexes and compounds from plants that have been previously used in ethnomedicine for the treatment of psoriasis.

## 3. Methodology

This narrative review aims to synthesize existing knowledge on the use of plant extracts and natural compounds in the treatment of psoriasis. A comprehensive literature search was conducted using electronic databases including PubMed, Elsevier, SpringerLink, Scopus, Web of Science, Google Scholar, and books on the subject. The search covered articles and the other relevant literature published up to May 2024. Keywords used in the search included combinations of terms such as “psoriasis”, “natural compounds”, “polyphenols”, “alkaloids”, “coumarins”, “terpenoids”, “plant extracts”, “traditional medicine”, “nanotechnology”, and “clinical studies”.

The selection criteria for this review included articles published in peer-reviewed journals, studies focusing on natural compounds with potential therapeutic effects on psoriasis, research articles, reviews, and clinical trials that investigated the mechanisms of action, efficacy, and safety of the compounds, as well as studies discussing the incorporation of these natural compounds into nanotechnological delivery systems.

The exclusion criteria were represented by articles that were not available in English, studies focusing on synthetic substances without reference to natural compounds, and papers that did not provide sufficient data on the mechanisms of action or clinical relevance of the compounds.

## 4. Plants Used in Traditional Medicine for Psoriasis

People have historically employed medicinal plants to treat a wide range of conditions, including dermatological and autoimmune diseases. Numerous herbal remedies are thought to either counteract some of the pathophysiological mechanisms of psoriasis or lessen the discomfort caused by the symptoms of the condition. Based on a number of Web of Science references, Table 1 lists the medicinal plants most frequently cited for their potential use in treating psoriasis.

Other plants used traditionally in psoriasis, especially in the southern region of Italy (Sicily) include *Acanthus mollis* (*Acanthaceae*), *Agave americana* (*Agavaceae*), *Artemisia arborescens* (*Asteraceae*), *Ecballium elaterium* (*Cucurbitaceae*), *Inula viscosa* (*Asteraceae*), *Parietaria officinalis* (*Urticaecae*), *Rhagadiolus stellatus* (*Asteraceae*), *Verbascum sinuatum* (*Scrophulariaceae*), and *Verbena officinalis* (*Verbenaceae*) [48].

## 5. Major Classes of Natural Compounds and Their Mechanisms of Action in Psoriasis

### 5.1. Alkaloids

Alkaloids found in certain plant sources exhibit different mechanisms in the treatment of psoriasis. These compounds exert their therapeutic effects through the modulation of inflammatory pathways, immune responses, and abnormal cell proliferation characteristic of psoriatic lesions. They may inhibit pro-inflammatory cytokines, such as TNF-α, interleukin-17 (IL-17), or interleukin-33 (IL-33), thereby reducing inflammation and suppressing the immune-mediated processes involved in psoriasis. Additionally, alkaloids can interfere with the proliferation of keratinocytes, the predominant cells involved in psoriatic plaque formation, by targeting pathways involved in cell division and growth. Some alkaloids also possess antioxidant properties, mitigating oxidative stress, which is known to exacerbate psoriasis. By targeting multiple pathophysiological aspects of psoriasis, natural alkaloids offer a promising approach for the development of novel therapeutic approaches for this chronic inflammatory disorder. The most well-known plant alkaloids that may have beneficial effects in psoriasis are listed below (Table 2).

### 5.2. Anthraquinones and Derivatives

These compounds are found abundantly in plants such as *Aloe vera* and *Rheum sp.* and possess promising properties that could prove useful in managing psoriasis. They exhibit anti-inflammatory properties by inhibiting key cytokines such as TNF-α, IL-6, IL-8, and IL-24, which play pivotal roles in the pathogenesis of psoriasis (Table 3) [119]. Additionally, these compounds demonstrate antioxidant activity, thus reducing oxidative stress, which is associated with exacerbating psoriasis. Furthermore, anthraquinones exhibit antimicrobial properties, potentially targeting infectious agents that trigger or aggravate psoriatic flare-ups [120]. Therefore, such compounds hold promise as adjunctive or alternative therapies in the management of psoriasis, offering potential benefits in reducing inflammation, suppressing hyperproliferation, and enhancing overall skin health.

### 5.3. Coumarins

The existing literature on coumarins and their implications in psoriasis highlights their versatile pharmacological properties and their potential as therapeutic agents. Studies have demonstrated that certain coumarins, including natural substances such as psoralen and esculetin, or their synthetic analogs (e.g., methoxsalen) exert anti-inflammatory effects by suppressing pro-inflammatory cytokines and modulating immune cell function [122,123,124]. For instance, esculetin significantly reduced pro-inflammatory cytokines such as TNF-α, IL-6, IL-22, IL-23, and IL-17A in psoriatic mouse skin and slowed down the disease’s progression [122]. Furthermore, coumarins interfere with key signaling pathways involved in keratinocyte proliferation and differentiation, thus restoring epidermal homeostasis in psoriatic lesions [125]. Additionally, the antioxidant properties of coumarins contribute to their efficacy by counteracting oxidative stress as a result of their capacity to scavenge reactive oxygen species (ROS) and prevent lipid peroxidation [126]. 

Psoralen plus ultraviolet-A radiation (PUVA) therapy was first introduced in the 1970s. Plant-derived photosensitizers referred to as psoralens are ingested or applied externally. The skin undergoes a therapeutically effective phototoxic response after exposure to UV-A radiation (315–400 nm). Given that response rates range from 74% to 100%, PUVA therapy represents an efficient therapy for psoriasis due to its anti-inflammatory and antiproliferative properties. However, it is less well tolerated compared to UV-B phototherapy (311 nm), and the phototoxic side effects of psoralen include nausea, headaches, and immune system suppression [127,128].

### 5.4. Flavonoids

Flavonoids, a diverse group of phytochemicals abundantly found in fruits, vegetables, and medicinal plants, have also garnered significant attention given their potential therapeutic effects. Several studies suggest that flavonoids exert their beneficial actions through multiple mechanisms, making them promising candidates for adjunctive therapy in psoriasis management [23]. 

Firstly, flavonoids possess significant antioxidant properties by scavenging ROS and reducing oxidative stress. By mitigating oxidative damage to skin cells and tissues, flavonoids may help alleviate symptoms associated with psoriasis flare-ups. Moreover, flavonoids exhibit anti-inflammatory effects by modulating various signaling pathways involved in the immune response [129]. For instance, they can inhibit the production of pro-inflammatory cytokines such as TNF-α, IL-1β, IL-6, IL-17, and IL-22, thereby attenuating the characteristic inflammatory cascade. Additionally, flavonoids have been shown to inhibit the activity of enzymes involved in inflammation, such as phospholipase A2, cyclooxygenases (COX), and lipoxygenases (LOX), which are involved in the biosynthesis of inflammatory mediators (e.g., prostaglandins and leukotrienes) [130]. Furthermore, representatives from this class possess immunomodulatory properties, regulating the function of immune cells, which plays a crucial role in psoriatic pathogenesis [131].

Some flavonoids have been found to promote regulatory T cell (Treg) differentiation while inhibiting the proliferation and activation of pro-inflammatory T helper (Th) 17 cells, thereby restoring immune balance, and reducing excessive inflammation. Additionally, flavonoids may exert antiproliferative and antiangiogenic effects on keratinocytes and endothelial cells, respectively [132,133].

The complex activities of flavonoids hold promise for the development of novel therapeutic strategies for psoriasis, offering potential benefits in terms of symptom relief, disease modulation, and an improvement in quality of life for patients. However, further clinical research is necessary to elucidate the specific mechanisms of action of different flavonoids and their optimal therapeutic dosages and formulations for the effective management of psoriasis. Some of the most important flavonoids that could prove beneficial in the adjunctive treatment of psoriasis are found in Table 4.

### 5.5. Phytosterols

Phytosterols have also generated interest considering their anti-inflammatory, immunomodulatory, antioxidant, and antiproliferative properties [156]. Studies indicate that phytosterols can suppress the production of pro-inflammatory cytokines, including TNF-α, IL-6, and IL-17, thereby mitigating the inflammatory response. In various immune cell models, phytosterols such as campesterol, β-sitosterol, and stigmasterol have demonstrated anti-inflammatory properties. Additionally, they have been shown to decrease the Th17 cell response and associated cytokines. Out of the three tested representatives, β-sitosterol presented the highest skin absorption and a clear beneficial activity in inhibiting inflammation, thus indicating its potential for development as an antipsoriatic agent [157]. Phytosterols also demonstrate immunomodulatory effects by modulating the function of various immune cells linked to psoriasis, leading to a reduction in the aberrant immune activation observed in this condition [158]. Moreover, phytosterols may also exert antiproliferative effects on keratinocytes, thereby contributing to the normalization of epidermal growth and differentiation in psoriatic skin.

### 5.6. Polyphenolic Acids

Since polyphenols have anti-inflammatory and immunomodulatory properties that may help control the immune response, they have been studied for their capacity to reduce psoriasis symptoms. Considering the polyphenolic acid subcategory, rosmarinic and caffeic acids prove essential in aiding psoriasis symptoms. These substances have been shown to block important inflammatory mediators (e.g., IL-1β, IL-6, IL-17, and TNF-α), which reduces the abnormal immune response (Table 5). Moreover, polyphenolic acids help restore cutaneous homeostasis by regulating many signaling pathways, including those involving NF-κB and MAPK, that have been linked to the etiology of psoriasis [159]. Consequently, the inclusion of polyphenolic acids in psoriasis treatment plans not only presents a viable approach to symptom relief but also highlights the capacity of natural substances to address complex molecular systems responsible for inflammatory and autoimmune disorders.

### 5.7. Polysaccharides

Natural polysaccharides derived from plant sources can exert therapeutic effects in the treatment of psoriasis through several mechanisms of action, given their immunomodulatory, anti-inflammatory, moisturizing, and tissue repair properties.

Certain polysaccharides have immunomodulatory properties. For example, polysaccharides from medicinal mushrooms like *Ganoderma lucidum* can modulate the immune function, increasing IL-10 and/or IL-1Ra expression, thus potentially reducing the autoimmune response that drives psoriasis [168]. Inflammation, on the other hand, is a key aspect of psoriasis symptoms such as redness, swelling, and itching. Natural polysaccharides also possess anti-inflammatory properties, which can help alleviate such symptoms. For instance, *Aloe vera* polysaccharides have been shown to inhibit pro-inflammatory cytokines and enzymes, thereby reducing inflammation in psoriatic skin lesions [169,170].

Since psoriasis is largely associated with dry, flaky skin due to impaired skin barrier function, polysaccharides with moisturizing properties can help hydrate the skin and improve its barrier function, reducing symptoms such as itching and scaling. For instance, β-glucans, structural components of plants, algae, and certain microorganisms, form a protective film on the skin, preventing moisture loss and enhancing skin hydration [171]. Their topical application promotes collagen deposition, reepithelialization, and tissue regeneration, which speeds the healing process of wounds [24]. Moreover, polysaccharides from *Aloe vera* and algae have also been shown to promote wound healing and tissue repair by stimulating collagen production, enhancing cell proliferation, and modulating inflammatory responses [172,173].

### 5.8. Terpenoids

Terpenoids are largely distributed secondary plant metabolites that possess anti-inflammatory, immunomodulatory, antioxidant, and antimicrobial properties that are relevant to psoriasis management (Table 6). Studies have demonstrated that terpenoids can inhibit pro-inflammatory cytokines (e.g., TNF-α, IL-1, IL-6, IL-8, IL-17, and IL-23), thus attenuating the inflammatory cascade. Moreover, terpenoids exert immunomodulatory effects by modulating the activity of immune cells connected to psoriasis pathogenesis, including T cells and dendritic cells [174]. Their antioxidant activity also helps mitigate oxidative stress, a contributing factor in psoriasis development. Furthermore, terpenoids exhibit antimicrobial effects against pathogens that exacerbate psoriatic lesions [175]. Overall, the existing data suggest that terpenoids hold great promise as potential therapeutic agents for psoriasis, though further research is needed to fully elucidate their mechanisms of action and optimize their clinical efficacy.

## 6. Clinical Studies on Natural Compounds or Plant Extracts for the Treatment of Psoriasis

There are few clinical studies performed in the last 10 years that assessed the efficacy of isolated natural compounds in the treatment of psoriasis and they are listed in Table 7. All these trials included patients with mild or moderate plaque psoriasis and consisted mostly of local treatments. The investigated natural compounds used as monotherapy in psoriasis were indirubin, curcumin, quercetin, limonene, genistein, cannabidiol, and tetrahydrocannabinol. Genistein and limonene were the only compounds tested orally. Just five of these trials were randomized, double-blind, had a medium duration of 8 to 12 weeks, and reported adverse effects. The main outcome measured was the Psoriasis Area and Severity Index (PASI) score. Indirubin [61], curcumin [189,190], cannabidiol [191], and genistein [192] proved to be more effective than the placebo in reducing PASI scores.

One study compared the efficacy and safety of different concentrations of indirubin in an ointment formulation and indicated that 200 μg/g of indirubin was the most effective dosage. Treatment with quercetin [193] and limonene [182] also showed promising results in reducing psoriatic lesions and symptoms, even though these studies were carried out on a small number of patients and without a control. Moreover, the successful use of topical cannabinoids (tetrahydrocannabinol) for the management of psoriasis was reported by Friedman et al. [194]. Although not detailed in this review, older studies described the use of berberine, capsaicin, and colchicine in the treatment of psoriasis as monotherapy [195,196].

**Table 7:** Summary of clinical studies performed with natural compounds as monotherapy.

Compound	Diagnosis	Study Design	Sample Size; Gender (M/F); Age: Mean ± SD	Treatment	Outcome Measure: Results	Dropouts; Adverse Events: (Incidence, Proportion of Group %)	Reference
Cannabidiol	Mild plaque-type psoriasis	Split-body, double-blind, randomized, placebo-controlled trial	51 patients; gender 30/21; mean age 53 years	Patients applied 2.5% cannabidiol ointment and a placebo twice daily for 12 weeks on their target plaques.	Statistically significant reduction in PASI and BSA scores after 2 weeks of treatment and over the total follow-up period.	Six patients had skin irritation and had to be terminated from this study. The AEs were not likely due to CBD, as they were observed equally on both sides.	[191]
Curcumin	Mild-to-moderate plaque psoriasis	Pilot randomized placebo-controlled trial	5 patients with at least two symmetrical skin lesions; gender 1/4; 38–52 years old	Patients applied a thin layer of 0.1% curcumin–niosome gel for one lesion and the placebo (gel base) for the counterpart, twice a day for 4 weeks.	Reduced redness, levels of psoriatic plaques, scaling, itching, and skin dryness. Significant downregulation of IL-17, IL-23, IL-22, TNF-α, S100A7, S100A12, and Ki67 in curcumin-treated lesions.	No AEs were reported. The curcumin–niosome suspension exerted low toxic effects on peripheral blood mononuclear cells from healthy donors, in an apoptosis assay.	[190]
Curcumin	Mild-to-moderate plaque psoriasis	Randomized, prospective intra-individual, right–left comparative, placebo-controlled, double-blind clinical trial	34 patients; gender 20/14; mean age 31.7 years	Patients applied microemulgel with 0.5% curcumin and a placebo (vehicle alone) on symmetrical lesions, twice daily, for 9 weeks.	The PASI score decreased on the side treated with the drug (redness, thickness, and scaliness of the lesions) and that was observed throughout the clinical trial (*p* < 0.05). The side treated with the placebo had a mild reduction in their PASI score up to week 3 and then remained almost the same all the way into week 9. Patients experienced a reduction in VAS (level of pruritus, burning, and pain) when using the turmeric microemulgel.	AEs: 6% of the patients reported dryness, 6% a burning sensation, and 3% irritation, identical for the left and right side, so they could not be associated with the product.	[189]
Genistein	Mild-to-moderate plaque psoriasis	Multicenter, randomized, double-blind, placebo-controlled, two-dose investigation	40 patients (30 drug, 10 placebo), age 30–62 years; 41% female and 59% male	15 patients (12 completed) received oral film-coated tablets with 75 mg genistein/daily, and 15 (12 completed) with 150 mg genistein, under fasting conditions, for 8 weeks.	Reduction in clinical (PASI, BSA, and PGA) and biochemical scores (TNF-α, IL-23).	6 (3 in each dose group) 42 AEs reported, of which the majority (78%) were mild (cold, herpes simplex, oral candidiasis, and right heel pain), and 22% were moderate (headache, sinusitis, rash, and pharyngitis).	[192]
Indirubin	Plaque psoriasis	Randomized, double-blind, four-arm, dosage-controlled trial	25 patients; gender 15/10 Age 36.7 ± 9.7	0.5 g ointment (200 μg/g of indirubin)/100 cm^2^ of the psoriatic lesion area, twice daily, topically for 8 weeks, follow-up at 20 weeks.	Reduction in PASI score: 69.2%, the proportion of subjects achieving 75 and 90% reductions in PASI scores: 56.5 and 30.4%.	2; AEs: 17, 70.8% non-related to trial medicine (nasopharyngitis, upper respiratory tract infection, mild erythema, eczema, allergic rhinitis, and asthma).	[197]
			25 patients; gender 16/9 Age 34.5 ± 8.8	0.5 g ointment (100 μg/g of indirubin)/100 cm^2^ of the psoriatic lesion area, twice daily, topically for 8 weeks, follow-up at 20 weeks.	Reduction in PASI score: 63.1%, the proportion of subjects achieving 75 and 90% reductions in PASI scores: 44 and 8%.	2; AEs: 8, 32% non-related to trial medicine (nasopharyngitis, upper respiratory tract infection, mild erythema, eczema, allergic rhinitis, and asthma).	
			25 patients; gender 18/7 Age 33.4 ± 7.3	0.5 g ointment (50 μg/g of indirubin)/100 cm^2^ of the psoriatic lesion area, twice daily, topically for 8 weeks, follow-up at 20 weeks.	Reduction in PASI score: 50.3%, the proportion of subjects achieving 75 and 90% reductions in PASI scores: 24 and 4%.	3; AEs: 16, 64% non-related to trial medicine (nasopharyngitis, upper respiratory tract infection, mild erythema, eczema, allergic rhinitis, and asthma).	
			25 patients; gender 11/14 Age 38.8 ± 10.9	0.5 g ointment (10 μg/g of indirubin)/100 cm^2^ of the psoriatic lesion area, twice daily, topically for 8 weeks, follow-up at 20 weeks.	Reduction in PASI score: 53.4%, the proportion of subjects achieving 75 and 90% reductions in PASI scores: 24 and 4%.	2; AEs: 19, 76% mostly non-related to trial medicine (nasopharyngitis, upper respiratory tract infection, mild erythema, eczema, allergic rhinitis, and asthma).	
Indirubin	Moderate plaque psoriasis	Randomized, double-blind, placebo-controlled study	24 patients (drug:16, placebo: 8); gender drug 10/6, placebo 7/1; mean age 39.6 ± 10.1	0.5 g ointment (0.24% indirubin) /100 cm^2^ of the psoriatic lesion area, twice daily, topically for 8 weeks, follow-up at 9 weeks.	In 56.3% of patients, the PASI scores were improved by 75%, and IL-17 in the skin genes of these patients was significantly downregulated compared with the placebo. Reduction in PGA score: drug (1.31 ± 0.6) compared to the placebo (2.29 ± 0.8).	1 placebo (at week 3) due to gout; AEs: placebo group 50.0% single events of pruritus, gout, allergies, and pyrexia; indirubin ointment group: 44.0% AEs: pruritus (25.0%), rash (12.5%), and nasopharyngitis (12.5%).	[61]
Indirubin	Mild-to-moderate plaque psoriasis	Preliminary in vivo test	3 patients	30 mg IN-PCL/PEO nanopatch covering over a surface area of 9 cm^2^ was applied topically with drops of water for 30 min, twice daily for up to 4 weeks. The patch contained 2.22% indigo, 0.33% indirubin, and 0.03% tryptanthrine.	Reduction in PASI score: a reduction in redness and scales was obtained after 2 weeks of application, with a noteworthy alleviation in erythema, infiltration, and scaling at the end of the 4-week treatment.	There was no regional irritation or discomfort reported over the entire treatment period, and no traces of dark stains on the skin or clothes were recorded.	[198]
Limonene	Mild-to-moderate plaque psoriasis	Pilot non-randomized study	9 patients; gender 3/6, age 13–73 years	Soft gel capsules with 222 mg orange peel extract containing 118 mg of *d*-limonene were given twice daily: topical (n = 5), oral (n = 1), or both (n = 3), for 45 days.	Reduction in clinical scores (PASI, VSACPSI, and NAPSI) and improvement of the objective DLQI.	No AEs were reported.	[182]
Quercetin	Mild-to-moderate stable chronic plaque psoriasis	Clinical trial	10 patients; gender 4/6; age 36.6 ± 13.01 years (18–58)	Patients were given a once-daily topical application of SCMC 3.5% gel containing a (0.02%) quercetin entrapped spanlastic formulation, for 8 weeks.	Statistically significant reduction in PASI score. The expression of livin was lowered while that of caspase-9 was elevated in tissue samples from patients after treatment.	Unobservable side effects (no irritation or sensitization on the application of the gel).	[193]
THC	Moderate plaque psoriasis	Case report	33-year-old, male	Tetrahydrocannabinol (THC) distillate cream, THC soap, 5 mg/mL, and a hair oil with THC distillate, 5 mg/mL, for 2 weeks with follow-up at 7 months.	After 2 weeks of use, the patient reported continued improvement and clearance. Seven months after initial use, the patient reported that he continues to use the products regularly (every few days) for maintenance.	No AEs were reported.	[194]

M—male; F—female; SD—standard deviation; AE—adverse events; PASI—Psoriasis Area and Severity Index, BSA—Body Surface Area, PGA—Physician’s Global Assessment; IN-PCL/PEO—*Indigo naturalis*-poly(ε-caprolactone,)/poly(ethylene oxide); VSCAPSI—Videodermoscopy Scalp Psoriasis Severity Index; NAPSI—Nail Psoriasis Severity Index; DLQI—Dermatology Life Quality Index; THC—tetrahydrocannabinol; SCMC—quercetin spanlastics formulation in sodium carboxymethylcellulose.

Phytochemicals are used in combination with synthetic drugs or phototherapy in order to control psoriasis symptoms and achieve long-term disease inactivity (Table 8). Most clinical trials focused on PUVA therapy which uses the sensitizing effects of psoralen combined with exposure of the skin to long-wave ultraviolet radiation in moderate to severe plaque psoriasis. All PUVA clinical trials used 8-methoxy psoralen taken orally, except for one retrospective hospital-based study that evaluated and proved the efficacy of topically applied psoralen in the form of a bath solution [199]. This route of administration can be used for patients who have contraindications to systemic immune-modifying drugs and are not responsive to narrowband (NB)-UVB phototherapy. Compared to broadband ultraviolet A (BB-UVA) therapy, PUVA significantly decreased PASI scores and had better clinical, immunohistochemical, and histopathological results [200]. Banerjee et al. [201] reported that PUVA is less effective than methotrexate in the treatment of extensive plaque psoriasis, with a higher incidence of subjective adverse effects, but with fewer laboratory complications (altered blood parameters). A comparison between PUVAsol therapy (oral psoralen plus sun exposure) and Unani therapy showed that they are equally efficient and well tolerated in patients with moderate to severe plaque psoriasis [202]. Another study demonstrated that the combination of PUVAsol with isotretinoin is more effective than PUVAsol alone for treating chronic plaque psoriasis [203].

Oral curcumin associated with topical methylprednisolone aceponate significantly decreased PASI scores in patients with mild to moderate psoriasis vulgaris and reduced serum levels of IL-22 compared to topical steroids alone [204]. In moderate to severe psoriasis, curcumin improved the outcome in patients treated with oral retinoids or phototherapy [205,206]. Thus, patients treated with acitretin plus curcumin NPs presented a higher decrease in PASI scores in contrast to patients who only received acitretin, and the combination therapy did not change the lipid profile [205]. Carrion-Gutierrez et al. [206] showed that oral curcumin activated with visible light phototherapy is as efficient as curcumin plus ultraviolet-A radiation, but it is safer for patients with moderate to severe plaque psoriasis. 

The addition of colchicine to secukinumab and guselkumab resulted in almost complete resolution of the skin lesions in two cases of generalized pustular psoriasis resistant to biologic monotherapy [207].

Shampoo formulations of cannabidiol [208] or salicylic acid [209] combined with other active ingredients showed promising results in scalp psoriasis. Topical application of salicylic acid and coal tar ointment was equally effective as calcipotriol/betamethasone dipropionate ointment after 12 weeks of treatment, although the latter caused a more rapid reduction in disease severity [210]. A balm containing celastrol in combination with the antipruritic agent polidocanol significantly decreased the symptoms of psoriasis when used alone or as an adjuvant to systemic drug therapy or phototherapy [211]. Celastrol is a pentacyclic triterpene from *Tripterygium wilfordii* and has the capacity to inhibit Th17/Th22 cytokine differentiation and factors triggered by IL-17, IL-22, and IFN-γ in keratinocytes [212]. 

Clinical trials that assessed the use of plant extracts in psoriasis in the last decade are described in Table 9, with the mention that Traditional Chinese Medicine formulations with a mixture of ingredients were not included in this review. The vast majority of trials evaluated the effects of plants on mild to moderate plaque psoriasis as topical applications. Only ten trials were randomized, double or triple-blind, seven were placebo-controlled, and three included positive controls. Most of them investigated plant extracts derived from *Indigo naturalis, Cannabis sativa,* and *Curcuma* sp., which all improved psoriasis lesions and symptoms. The concentration of active compounds in extracts is mentioned for merely three studies: indirubin levels in the oily extract of *Indigo naturalis* [213], α- and β-santalol concentration in *Santalum album* volatile oil [214], and cannabigerol–cannabidiol concentration in *Cannabis* oil [215].

Standardization in herbal medicine is needed to ensure the safety and quality of the vegetal product, and the reliability and predictability of therapeutic effects. While clinical trials with non-standardized herbal medicines have significant limitations, they can still be useful, particularly in the early stages of research. 

Among the studied extracts, orally administered *Tripterygium wilfordii* extract stands out for its activity comparable to that of acitretin in the treatment of moderate to severe psoriasis [216]. *Tripterygium wilfordii* has been used for centuries in China to treat immune diseases, inflammation, and tumors, and numerous clinical trials that support its efficiency in the treatment of psoriasis are examined in a meta-analysis by Lv et al. [217]. Moreover, a retrospective analysis was conducted for children from southwestern China aged 14 years old or less with the von Zumbusch type of generalized pustular psoriasis: 11 patients were treated and responded to *Tripterygium wilfordii* Hook F (TwHF), together with acitretin or not [218]. 

In some of the studies, plant extracts are used in combination with other compounds, plants, or therapies: *Aloe vera* plus propolis [219], *Kunzea ambigua* essential oil plus salicylic acid and liquor carbonis detergens [220], and *Curcuma longa* hydro-alcoholic extract plus visible blue light phototherapy [221]. In the last study, *Curcuma longa* extract combined with visible blue light phototherapy proved to be as effective as PUVA and safer for patients with moderate to severe plaque psoriasis, even if the speed of this improvement may be slower than that of PUVA treatment [221].

In conclusion, there are few clinical trials that evaluate the effect of natural compounds or plant extracts in psoriasis and their value is limited by the following factors: the small number of patients, insufficient to reach statistical significance; the lack of a positive control or placebo; the short duration of studies and the wide variation of time periods; a lack of follow-up or recurrence reported in many studies; the focus on psoriasis vulgaris (usually mild-to-moderate), ignoring other forms of the disease; the scarcity of toxicological and safety evaluations and the lack of standardization of plant extracts. Moreover, half of the reviewed studies are not randomized and double-blind, making their results more likely to be biased.

**Table 8:** Summary of clinical studies performed with natural compounds in combination therapy.

Compound	Diagnosis	Study Design	Sample Size; Gender (M/F); Age: Mean ± SD	Treatment	Outcome Measure: Results	Dropouts; Adverse Events	Reference
Cannabidiol	Mild-to-moderate scalp psoriasis or seborrheic dermatitis	Clinical study	50 patients; 22 subjects had scalp psoriasis and 28 subjects had seborrheic dermatitis; gender 24/26; 18–61 years (mean age 38 and 46 years, respectively).	Shampoo containing 0.075% broad-spectrum cannabidiol, ketoconazole, and numerous ingredients that promote hair growth, used for 2 weeks without changing the shampooing frequency.	Severity scores for erythema and scaling, itching, and burning reduced significantly. Reduction in severity scores for arborizing vessels, twisted capillaries, and scales.	No AEs were reported. Subject assessment mean scores for tolerability and overall satisfaction were both 9 out of 10.	[208]
Celastrol	Mild-to-moderate body plaque psoriasis	Monocentric open clinical studies	94 patients; 43 patients (celastrol balm alone), gender 20/23, mean age 52; 51 patients (balm in association with topical or systemic drug treatments or phototherapy), gender 28/23; mean age 49.6.	Once-a-day application of celastrol balm over the whole body, for 4 weeks. The balm is a thick oil/water emulsion with 0.3% celastrol-enriched extract and 1% polidocanol. The celastrol-enriched extract is a well-defined, triptolide-free extract.	In the ”balm alone” study: statistically significant (*p* < 0.001) decrease in the mean pruritus intensity score and in the mean dryness score of psoriasis plaques at day 8 (−39%, −47%), and day 29 (−60%, −46%). In the ”balm in association” study: significant (*p* < 0.05) decrease in desquamation, dryness, pruritus, and “tightness”.	2, respectively, 1 dropout per study. No AEs related to the product were reported.	[211]
Salicylic acid	Mild to moderate psoriasis	A single-center, randomized, double-blind, vehicle-controlled study	67 adult patients; 65.7% female; mean age 43.2 years.	The active shampoo or its vehicle was applied daily for 14 days and 3 times/week for another 14 days. The keratolytic and hydrating shampoo contained 2% salicylic acid, 5% urea, and 1% glycerin.	The active shampoo significantly reduced the PSSI score by 39.0%, 37.2%, 63.0%, and 69.0% immediately after washing compared to a 22.8%, 5.5%, 19.6%, and 13.0% with the vehicle at days 1, 8, 15, and 30, respectively. SCALPDEX items, IGA, and irritation were significantly reduced with the active shampoo.	No AEs were reported. The shampoo was well tolerated and subjects were highly satisfied and had an improved quality of life.	[209]
Salicylic acid	Limited chronic plaque psoriasis	Prospective open-label randomized trial	62 patients; gender and age non-specified	33 patients (20 completed) received a topical application of 6% coal tar with 3% salicylic acid ointment (group A) and 29 patients (20 completed) received calcipotriol/betamethasone dipropionate (group B), once at night for 12 weeks.	The reduction in PASI score at week 12 was 61.5% in group A vs. 40.7% in group B. A PASI score of 50 was attained by 8 of 20 patients (40%) in group A compared to 9 of 21 patients (42.8%) in group B. One case (5%) in group A and 4 (19%) in group B had complete clearance of the disease.	No AEs were observed in the 2 groups.	[210]
Colchicine	Generalized pustular psoriasis	Case report	2 patients: a 48-year-old woman (patient 1) and a 28-year-old woman (patient 2)	Patient 1 was started on secukinumab, and patient 2 was started on guselkumab, both showing initial improvement at 3 to 4 weeks then worsening or recurrence at week 6 to week 8. At the time of worsening, both women were started on colchicine 1.5 mg daily in addition to continuing current biologic therapy.	By week 20 (patient 1) and week 16 (patient 2), there was almost complete remission for both, respectively.	No AEs were reported.	[207]
Curcumin	Mild-to-moderate psoriasis vulgaris	Randomized, double-blind, placebo-controlled clinical trial	63 patients; gender 14/17 (arm 1) and 18/14 (arm 2); mean age 37 (arm 1) and 41 (arm 2)	31 patients were treated with topical methylprednisolone aceponate and oral Meriva (commercially available lecithin-based delivery system of curcumin) at 2 g per day (arm 1), and 32 patients with topical methylprednisolone aceponate alone (arm 2) for 12 weeks. 4-week follow-up period.	Both groups achieved a significant reduction in PASI values at week 12 that, however, was higher in patients treated with both topical steroids and oral curcumin. At the follow-up visit, week 16, the reduction in PASI values remained significant for both groups, but still higher for the first group. IL-22 serum levels were significantly reduced only in patients treated with oral curcumin. No significant changes in IL-17 levels were reported in both groups.	6 dropouts (arm 1) and 8 dropouts (arm 2); AEs: 1 patient treated with Meriva (diarrhea); 2 patients treated with placebo (papular eruption on the face and nausea).	[204]
Curcumin	Moderate-to-severe psoriasis	Placebo-controlled, double-blind, randomized clinical trial	30 patients; 7/8; 24–63 years (arm 1) and 9/6; 29–59 years (arm 2)	15 patients were treated orally with acitretin (0.4 mg/kg per day) plus nanocurcumin (3 g/day), and 15 patients with acitretin plus placebo, for 12 weeks. Follow-up at 4 weeks.	The reduction in PASI score was significantly higher in patients treated with curcumin (*p* < 0.0001) and remained significant at week 16. At week 12, a PASI score of 50 was achieved by 13 patients in arm 1 (93%) and 10 in arm 2 (66%). A PASI score of 75 was reached in 6 patients in arm 1 (43%) and 3 in arm 2 (20%). A PASI score of 90 was achieved by 5 patients in arm 1 (36%) and two in arm 2 (13%). Cholesterol serum levels remained unchanged in patients treated with acitretin plus nanocurcumin.	AEs: 1 patient treated with curcumin reported nausea and vomiting at week 3, and abandoned the trial. 7 (arm 1) and 9 (arm 2) reported reversible effects of retinoids (mild cheilitis and peeling of the palms and soles).	[205]
Curcumin	Moderate-to-severe psoriasis	Randomized, double-blind, placebo-controlled, pilot clinical trial	21 patients; gender 13/8; age 41.3 ± 12.0 years	Oral curcumin with local phototherapy (VLRT—11 patients or VLST—10 patients) in the experimental area, while the rest of the body surface was treated with UVA. 48–72 h before starting phototherapy, patients began taking the tablets daily (100 mg of standardized *Curcuma longa* extract with 12 mg of curcumin per tablet) and the treatment took place twice a week for 2 months.	81% of the patients in the VLRT group and 30% of the patients in the VLST group showed an evolution of their lesions in the selected area to at least “Slight”, whereas 76% of all patients in both groups showed the same evolution of the lesions on the rest of the body surface (UVA).	There were no study-related adverse events that necessitated participant withdrawal.	[206]
Methoxy-psoralen	Chronic plaque psoriasis	Randomized, hospital-based study	40 patients; gender 16/4, mean age 40.55 (group A) and gender 13/7, mean age 35.65 (group B)	20 patients (group A) received PUVAsol only; 20 patients (group B) received PUVAsol + isotretinoin (0.5 mg/kg/day) for 12 weeks. PUVAsol = 8-methoxypsoralen (0.6 mg/kg) taken orally in the morning, followed by sunlight exposure after an interval of 2 h on three alternate days in a week.	12.5% and 37.5% of patients in group A achieved PASI scores of 75 and 50, respectively, after a mean duration of 10 weeks. In group B, 63.15% and 84.21% of patients achieved PASI scores of 75 and 50, respectively, in a mean duration of 8 weeks. At week 12, none of the patients in group A achieved a PASI score of 90, but 31.57% of patients in group B did. DLQI scores significantly improved after 12 weeks in both groups.	4 dropouts (group A) and 1 (group B). AEs in group A: 12 patients (nausea, hyperpigmentation, erythema, and pruritus). AEs in group B: 14 patients (cheilitis, nausea, erythema, and pruritus).	[203]
Methoxy-psoralen	Moderate-to-severe psoriasis	Retrospective, hospital-based study	120 patients; gender 96/24; mean age 53.4 years (32–83)	Bath solutions were prepared at a 0.0003% (wt/vol) concentration of 8-methoxypsoralen. The skin was soaked for 15–20 min and then quickly wiped dry. Immediately afterward, patients were exposed to UVA radiation.	The average PASI scores decreased from 20.8 ± 7.9 to 5.1 ± 5.4. 67 patients (69.7%) achieved at least a PASI score of 75 and, of them, 38 (39.6%) had a PASI score of 90.	24 dropouts; 30 AEs were reported: 24 phototoxic burns and 6 itching. AEs were mild and transitory.	[199]
Methoxy-psoralen	Chronic plaque psoriasis	Randomized, controlled clinical trial	61 patients; Group IA: gender 14/2; mean age 43.81c14.01 Group IB: gender 8/7; mean age 46 ± 12.30; Group II: gender 15/15; mean age 41.63 ±13.97	Group I (n = 31) was randomized to either IA or IB who received BB-UVA 10 (n = 16) or 15 J/cm^2^ (n = 15) per session, respectively, while group II (n = 30) received PUVA with 8-methoxypsoralen, 0.7 mg/kg, waiting two hours before exposure to UVA. Therapy was delivered thrice weekly until clearance or 48 treatments at most.	Clearance of psoriasis: 31.25% (IA), 33.3% (IB), and 76.7% (II). PASI scores were reduced within each group. The UVA group achieved results comparable to PUVA until session 24 but failed to match it at the final evaluation. Both treatments caused a reduction in dermal lymphocytic counts and epidermal bcl-2 expression.	IA: 2 dropouts; 2 AEs (phototoxic reaction and pruritus); IB: 1 dropout; 2 AEs (phototoxic reaction) II: 4 dropouts; 6 AEs (pruritus and nausea).	[200]
Methoxy-psoralen	Severe chronic stable plaque psoriasis	Randomized, open-label clinical study	60 patients; Methotrexate group: gender 18/12; mean age 43.26 ± 13.16. PUVA group: gender 20/10; mean age 40.33 ± 14.9	30 patients received methotrexate at a dose of 0.4 mg/kg up to a maximum of 15 mg/week and the other 30 were treated with PUVA, 8-methoxy psoralen tablet (20 mg) followed by UVA. Both forms of treatment were continued for 10 weeks or until a PASI score of 90 was achieved, whichever was earlier.	In the PUVA group, 90% achieved PASI-50 and 63.33% achieved PASI-90, while in the methotrexate group 100% patients achieved both PASI-50 and PASI-90. Methotrexate acted significantly faster than PUVA in disease clearance. All patients of the methotrexate group achieved PASI-90 after 6.17 ± 1.42 weeks with a mean dose of 100.00 ±21.24 mg. In the PUVA group, 63.33% patients achieved PASI-90 after 9.11 ± 0.81 weeks.	AEs in the PUVA group: 43.33% dryness and itching; 40% grade 1 erythema; 33.33% nausea and vomiting; 16.67% pigmentation; 13.33% exacerbation; and 3.33% elevated bilirubin. AEs in the methotrexate group: 13.33% decreased platelet count; 10% decreased hemoglobin; 10% elevated liver enzyme; and 3.33% decreased leucocyte count.	[201]
Methoxy-psoralen	Chronic plaque psoriasis	Randomized, controlled clinical trial	287 patients; PUVAsol therapy: 140, gender 101/39, mean age 37.2 ± 13.7 Unani treatment: 147, gender 118/29; mean age 37.3 ± 11.5	PUVAsol group: patients were given 8-methoxy-psoralen (0.6 mg/kg) orally on alternate days as a single dose after breakfast, followed 2 h later by sun exposure (5 increasing to 30 min). Unani group: patients took two capsules of 500 mg UNIM-401 orally twice a day and applied UNIM-403 oil on the lesions once a day, followed 2 h later by exposure to sunlight. 12 weeks treatment and follow-up at 12 weeks. UNIM-401 is the dried aqueous extract of *Fumaria parviflora*, *Swertia chirata*, *Psoralea corylifolia,* and *Terminalia chebula* (ratio 5:5:5:2). UNIM-403 contains the inner bark of *Azadirachta indica* and *Cinnamomum camphora* dispensed in sesame oil.	15.7% of patients in the PUVA sol group and 16.3% in Unani group achieved a PASI score of 75. 65.2% patients in the PUVA sol group and 71.4% in the Unani group achieved a PASI of 50. The proportion of patients who relapsed at 24 weeks was comparable: 1 in the PUVAsol group and 3 in the Unani therapy group.	41 (and 39, respectively) patients were lost to follow-up and 32 (and 24, respectively) patients were withdrawn. AEs: 16.4% of patients in the PUVA sol group (gastro-intestinal symptoms, headache, palpitation, and phototoxicity) and 2% of patients in the Unani group (gastro-intestinal symptoms).	[202]

M—male; F—female; SD—standard deviation; AE—adverse events; PASI—Psoriasis Area and Severity Index; VLRT—real visible light phototherapy; VLST—simulated visible light phototherapy; UVA—ultraviolet A radiation; PSSI—psoriasis scalp severity index; IGA—investigator’s global assessment; SCALPDEX—scalp dermatitis–specific quality-of-life instrument; PUVAsol—oral psoralen + sun exposure; DLQI—Dermatology Life Quality Index; BB-UVA—broadband ultraviolet A.

**Table 9:** Summary of clinical studies performed with plant extracts.

Plant	Diagnosis	Study Design	Sample Size; Gender (M/F); Age: Mean ± SD	Treatment	Outcome Measure: Results	Dropouts; Adverse Events: (Incidence, Proportion of Group %)	Reference
*Aloe vera* + propolis	Mild-to-moderate psoriasis	Double-blind, placebo-controlled study	2248 patients	Group 1 was treated with an ointment containing propolis 50% and *Aloe vera* 3%. Group 2 was treated with a placebo (ointment without propolis and *Aloe vera*). Topical treatment was performed for 12 weeks.	In Group 1: cleared in 64.4% of cases (excellent response), good response in 22.2%, weak response in 5.6%, and no response in 7.7%. In Group 2 (placebo group), no significant improvement was observed after 12 weeks of treatment. Histology demonstrated a marked reduction in hyperkeratosis and acanthosis.	No severe side effects were noted. Minimal discomfort due to the texture of the ointment and a temporal itching sensation were observed.	[222]
*Aloe vera* + propolis	Mild-to-moderate palmoplantar psoriasis	Clinical trial	857 patients; gender 503/354; age 9–62	All patients were treated with an ointment containing propolis 50% and *Aloe vera* 3% for 12 weeks.	86% of patients had a reduction in PASI scores; 62% of them showed excellent results and 24% showed good results.	No AEs were reported.	[219]
*Boswellia serrata* resin extract or *Vaccinium myrtillus* seed oil	Psoriasis or erythematous atopic eczema	Randomized, placebo-controlled, double-blind study	59 patients; gender 33/26; mean age 34.46; divided in 3 groups, each containing 10 psoriasis patients and 10 patients with erythematous dermatitis (except the Bosexil^®^ group which included 9 dermatitis patients)	Patients were randomly assigned, in a 1:1:1 ratio, to Bosexil^®^ (*Boswellia serrata* resin extract), *Vaccinium myrtillus* seed oil, or the placebo, topically applied. Patients applied the cream twice a day to the affected areas, for a period of 30 days.	“Change of condition”: patients with psoriasis, scales, and erythema improved with both treatments in comparison with the placebo. The Bosexil^®^ formulation improved scales (70% of cases) and erythema (50% of cases) without any cases of worsening. The *V. myrtillus* seed oil formulation improved erythema in 10% of cases, scales in 80% of cases, and 30% of patients were in remission at the end of therapy. PASI scores decreased with active treatments.	No AEs were reported.	[223]
*Cannabis sativa* CBD-enriched ointment	Moderate-to-severe psoriasis, atopic dermatitis, and resulting outcome scars	Retrospective study	20 patients: gender 6/14, age 20–80. Distribution of patients: psoriasis (5), atopic dermatitis (5), and resulting outcome scars (10)	The subjects applied topical CBD-enriched ointment to lesioned skin areas twice daily for three months. The ointment contained CBD seed oil, *Mangifera indica, Calendula officinalis, Lavandula officinalis,* chamomile, *Amyris balsamifera,* and shea butter	Skin parameters (hydration, transepidermal water loss, and elasticity) significantly improved. A reduction in the numbers of papules (−20%) and pustules (−31%) was noted. PASI scores were significantly reduced (*p* < 0.001) at day 90.	No irritant or allergic reactions were documented.	[224]
* Cannabis sativa * CBG/CBD oil	Psoriasis	Patent	2 patients; gender and age non-specified	Each subject received topical application of a thin layer of 3% CBG/CBD oil on the upper lesion and 15% CBG/CBD oil on the lower lesion of the left arm and a placebo (0% CBG/CBD oil) on the two lesions of the right arm, twice daily for 6 weeks. The ratio of CBG:CBD in the oil was 2:1.	The 3% CBG/CBD oil treatment showed no improvement in the lesions. The 15% CBG/CBD oil treatment showed 16% improvement in subject 1 and 33% improvement in subject 2 with psoriasis vulgaris.	No AEs were reported.	[215]
*Curcuma longa*	Mild-to-moderate scalp psoriasis	Randomized, double-blind, placebo-controlled, prospective clinical trial	40 patients enrolled / 30 completed: 15 (drug), gender 6/9, 29 years; 15 (control), gender 3/12, 44 years;	The case group received turmeric tonic twice a day for 9 weeks, whereas the other group received a placebo applied in the same manner.	Compared to the placebo, turmeric tonic significantly reduced the erythema, scaling, and induration of lesions (PASI score), and also improved patients’ quality of life measured by DLQI scores (*p* value < 0.05).	2 placebo patients complained about dryness and withdrew, and 8 patients were lost to follow-up. No AEs from the turmeric tonic were reported during the study, and at least for 4 weeks post-trial.	[225]
*Curcuma longa* hydro-alcoholic extract + visible blue light phototherapy	Moderate-to-severe psoriasis	Randomized, with third party blind evaluation, unicenter, open pilot clinical trial	24 patients	Participants were randomized to receive 600 mg/day of *Curcuma* extract and visible blue light or conventional PUVA therapy with methoxsalen, for 12 weeks.	11 (85%) out of 13 in the *Curcuma* group and 10 (91%) out of 11 in the PUVA group showed a PASI score reduction equal to or higher than 75% at the end of the study. The speed of response seemed to be slower in the *Curcuma* group (61 ± 6 vs. 42 ± 4 days).	AEs in *Curcuma* group: rare and mild; In the PUVA group, patients required sun protection, and some of them showed gastric distress.	[221]
*Curcuma xanthorrhiza*	Mild psoriasis	Randomized, double-blind, intra-individual, controlled trial	17 patients; gender 13/4; 18 to 59 year-old, with 2 similar lesions on the body	Lesions were treated with 1% *C. xanthorrhiza* ointment vs. a placebo (containing vaselinum album, the vehicle of the *C. xanthorrhiza* ointment) for 4 weeks.	PASI scores were reduced significantly in both lesions, either treated with 1% *C. xanthorrhiza* ointment or the placebo. The Trozak score was reduced in lesions treated with 1% *C. xanthorrhiza* ointment but increased significantly in lesions treated with the placebo. There was no significant difference of K6 expression before and after treatment in both groups.	No AEs were reported.	[226]
*Gracilaria* algae extract	Mild-to-moderate plaque-type psoriasis	Triple-blinded, randomized, body-split, controlled clinical trial	30 patients (12/18; 41.36 ± 14.09) with 94 symmetrical psoriasis plaques	Patients received either Clobetasol cream 0.05% or *Gracilaria* algae cream 3% on right or left-sided symmetric plaques once daily for 8 weeks and follow-up at 4 weeks.	The modified PASI score was reduced more on the sides treated with *Gracilaria* algae cream (0.80 ± 0.19% vs. 0.63 ± 0.25%, *p* < 0.05). No significant difference was found regarding the mean PGA scores between the 2 groups (*p* > 0.05). Patients’ satisfaction using VAS scores was significantly higher in favor of algae cream only at week 8.	In both algae and Clobetasol cream, 5 patients reported slight pruritus in the first 4 weeks of intervention that resolved spontaneously.	[227]
*Hypericum perforatum*	Mild-to-moderate plaque-type psoriasis	Double-blind, placebo-controlled, pilot study with intra-individual comparison	12 patients with lesions on both sides of the body; gender 4/8; 18–55 years old	*H. perforatum* ointment and a placebo (vehicle) were applied on symmetrical lesions on the body, twice daily for 4 weeks. The formulated ointment was prepared from an extract of *H. perforatum* L (5% wt/wt), vaseline (84% wt/wt), propylene glycol (10% wt/wt), and avicel (1% wt/wt).	*Hypericum* ointment significantly lowered PASI scores (erythema, scaling, and thickness) compared to the placebo (*p* = 0.014, *p* = 0.004, and *p* = 0.003, respectively). Significant improvement in clinical and histological (spongiosis, acanthosis, parakeratosis, hypogranulosis, thinning of suprapapillary plates, and Munro microabscesses) features of treated lesions in comparison with the placebo was observed (*p* < 0.05). TNFα concentrations in the dermis, endothelial cells, and dendrite cells were significantly reduced in lesions treated with the drug.	No report of allergic reactions and side effects for both drug and placebo ointment. One patient had a recurrence of the lesions.	[228]
*Indigo naturalis* extract in oil (Lindioil)	Nail psoriasis	Randomized, observer-blind, vehicle-controlled trial	31 patients with symmetrically comparable psoriatic nails; gender 24/7, mean age 40.7 ± 12.6 years	Lindioil or olive oil (control group) was applied topically to the same subjects’ two bilaterally symmetrical psoriatic nails twice daily for the first 12 weeks and then subjects applied Lindioil to both hands for 12 additional weeks. Lindioil = powdered leaves of *Baphicacanthus cusia* mixed with olive oil (1:10). The indigo naturalis powder used contained 3.15% indigo blue and 0.15% indirubin. The final concentration of indirubin in Lindioil was adjusted to 200 µg/g.	The reduction in NAPSI scores for the 12-week treatment for the Lindioil group (49.8% for one hand and 59.3% for a single nail) was superior to the reduction in the scores for the control group (22.9% and 16.3%, respectively). The application of Lindioil on both hands beginning at week 13 resulted in similar clinical effects at week 24. At week 12, the Lindioil group had better SGA scores than the olive oil group (2.4 ± 1.1 vs. 1.2 ± 1.2, *p* < 0.001) and had better PGA scores (2.8 ± 1.2 vs. 1.2 ± 1.1, *p* < 0.001). The positive-response rate was significantly higher in the Lindioil group than in the control group (60% vs. 13.3%).	30 subjects (96.8%) remained in the study at week 12 and 27 subjects (87.1%) remained at week 24. There were no AEs noted such as pain, itching, or erythema around the nail folds during the 24 weeks of treatment.	[213]
*Indigo naturalis* and *Indigofera tinctoria*	Chronic plaque psoriasis	Randomized, double-blind pilot study	54 patients were divided into three groups to receive either Iranian herbal ointment (18; 9/9; 41.5 ± 10.98), Chinese herbal ointment (18; 9/9; 33.08 ± 10.92), or betamethasone 0.1% ointment (18; 7/11; 31.6 ± 8.98)	Patients applied the ointments twice daily for 8 weeks. The Chinese herbal ointment was prepared from 20% *Indigo naturalis* powder and 80% vehicle (25% vaseline, 30% yellow wax, and 45% olive oil). The Iranian ointment was prepared from 20% *Indigofera tinctoria* extract (plant powder with 70% ethanol) and 80% of the same vehicle.	The mean PASI score change from the baseline was statistically significant only in the topical betamethasone group (from 7.08 ± 2.72 to 3.09 ± 2.23). For the Iranian and Chinese herbal ointments, PASI scores decreased only slightly (from 6.45 ± 2.36 to 6.32 ± 2.85, and from 6.86 ± 2.3 to 5.8 ± 3.13, respectively).	AEs: dermatitis in 7 patients of the Iranian herbal ointment group, and 5 in the Chinese herbal ointment group. In each herbal ointment group, 1 patient experienced severe dermatitis, which needed treatment and led to discontinuing the intervention. 7/7/8 patients from each group were lost to follow-up.	[229]
*Kunzea ambigua* essential oil + salicylic acid + LCD	Mild-to-moderate psoriasis	Randomized, comparative, double-blind study	30 patients (gender 12/18; mean age 52.8 ± 13.6 years)	15 patients received ointment and/or scalp lotion containing 20% kunzea oil (test group) and 15 patients received control medications not containing kunzea oil (control group). Formulations in both treatment arms also contained 5% liquor carbonis detergens (LCD) and 3% salicylic acid.	Both test and control groups demonstrated a significant improvement in PASI scores: the test group had a decrease from 12.7 to 6.7, whereas the control group showed a decrease from 8.1 to 3.5. No statistically significant difference was observed between treatment regimens. The VAS score of pruritus decreased by 77% for the test group and 72% for the control group. VAS scoring for scalp formulation displayed a statistically comparable improvement rate of 73% in the test group compared with 54% in the control.	No serious AEs were reported. 4 subjects (3 in the test group and 1 in the control group) reported itchiness after applying the scalp lotion, only for several days at the start of the treatment. One patient in the test group complained about the odor of the formulation and subsequently withdrew from the trial.	[220]
*Nigella sativa*	Mild-to-moderate plaque and palmoplantar psoriasis	Randomized clinical trial, open-label, therapeutic, outpatient-based	60 patients; 28 males with a mean age of (35.7 + 11.9) years, 32 females with a mean age of (35.3 + 12.9) years	20 patients (group 1) were treated with 10% (*w*/*w*) ointment of NS, 20 patients (group 2) were treated with crude powder of NS, 500 mg capsules three times daily; 20 patients (group 3) were treated with the combination of ointment and capsules of NS. Treatment for 12 weeks, plus follow-up at 4 weeks.	Reduction in PASI scores in group 1: from 9.0 ± 3.7 to 4.3 ± 2.0, group 2: from 9.9 ± 3.4 to 5.4 ± 2.7, and group 3: from 10.9 ± 2.7 to 4.2 ± 1.7. The clinical response to treatment after 12 weeks: an excellent response and a good response were noted in 85% of patients (group 3), while it was 65% and 50% of patients in groups 1 and 2, respectively. The disease relapsed in 31%, 50%, and 18% of patients on the ointment, capsules, and the combination, respectively. The serum level of malondialdehyde was significantly decreased only in groups 1 and 3.	No AEs were observed.	[230]
*Santalum album* volatile oil	Mild-To-moderate plaque psoriasis	Single-center, open-label safety, tolerability, and efficacy clinical trial	12 adult subjects	The study medication was topically applied twice a day for 28 days and consisted of an anhydrous serum formulation of EISO (10% *w*/*w* EISO in a caprylic/capric triglyceride, dimethyl isosorbide ethoxydiglycol formulation). The concentrations of α- and β-santalol in EISO were 49.0% and 20.8%.	In 9 of the 11 evaluable subjects, the severity of psoriatic plaques was reduced by the end of the study. Overall, 64% of the subjects (7/11) demonstrated a reduction in their IGA score during the 28-day treatment period. EISO treatment of the psoriasis skin model reverted psoriatic pathology as demonstrated by histologic characterization and the expression of keratinocyte proliferation markers Ki67 and psoriasin. It also suppressed the production in pro-inflammatory cytokines ENA-78, IL-6, IL-8, MCP-1, GM-CSF, and IL-1b.	One patient withdrew from the study with a mild adverse event after 3 weeks. The mild skin reaction at the application site resolved upon withdrawal.	[214]
*Tripterygium wilfordii*	Moderate-to-severe psoriasis vulgaris	Randomized, double-blind, double-dummy, parallel-group clinical study	115 patients enrolled (58 in TwHF group, gender 38/20, 42.0 ± 12.0 years, and 57 in the acitretin group, gender 43/14, 40.2 ± 12.4 years)	Patients received either a chloroform–methanol extract of TwHF 20 mg, 3 times a day plus a placebo matching acitretin 30 mg once a day or acitretin 30 mg once a day plus a placebo matching TwHF 20 mg 3 times daily, both for 8 weeks.	The median PASI score improved in the TwHF group by 50.4% and in the acitretin group by 42.7%, with no significant difference between the two groups at 2, 4, and 8 weeks. There was also no significant difference in PASI-25, PASI-50, PASI-75, and PASI-90 response between the two groups at 2, 4, and 8 weeks. There was a significant increase in the level of aspartate transaminase and triglycerides in the TwHF group, and a significant increase in the level of alanine transaminase, cholesterol, and high-density lipoprotein in the acitretin group.	Discontinuation occurred because of withdrawn consent (1), loss to follow-up (8), an AE (1), and disease progression (2). More AEs were reported in the acitretin group (78.1%, dry mucosa, facial pigmentation, hair loss, paronychia, and palpitation) compared with the TwHF group (43.6%, menstrual disorders, dry mouth, gastrointestinal complaints, and swelling of the lower limbs).	[216]
Traditional herbal supplement	Mild-to-severe psoriasis	Retrospective clinical case study	3 patients; female 17 years, mild psoriasis; male 50 years, moderate psoriasis; male 31 years, severe psoriasis;	2 capsules of Taraxaf (consisting of dandelion, olive leaf, nettle leaf, and turmeric in equal quantities; each capsule is 1 g), with 2 capsules of milk thistle (2 g) daily, half an hour before breakfast, for 5 months	The severity index scores were 3.2, 14, and 16.2, respectively, and improved posttreatment to 0, 0.8, and 2, respectively. The female showed complete healing after 3 months.	No AEs were reported.	[231]

M—male; F—female; SD—standard deviation; AE—adverse events; PASI—Psoriasis Area and Severity Index; PGA—Physician Global Assessment; VAS -Visual Analogue Scale; CBD—cannabidiol; k6—marker of hyperproliferative, activated keratinocytes, found in inflammatory disorders; Trozak—histological assessment score of psoriasis vulgaris; NAPSI—Nail Psoriasis Severity Index; SGA—subject global assessment; LCD—liquor carbonis detergens, an alcoholic solution of crude coal tar; EISO—East Indian Sandalwood oil (*Santalum album* essential oil); ENA78—epithelial neutrophil-activating peptide 78; IGA—Investigator Global; Assessment; DLQI—Dermatology Life Quality Index; TwHF—extract of *Tripterygium wilfordii* HookF; CBD—cannabidiol; CBG—cannabigerol; NS—*Nigella sativa*.

## 7. Nanotechnological Approaches for the Treatment of Psoriasis

In order to increase the effectiveness of substances through a higher release profile, better skin penetration, and a reduction in the adverse effects of conventional formulations, studies were directed toward the field of nanotechnology. 

There are several advantages of nanotechnology in the local treatment of psoriasis, out of which the following can be mentioned: protection of the active substance with the use of various nanoformulations [232], a high concentration of the active substance through the film created on the surface of the skin or by deposition in the lipid matrix of the stratum corneum and thus a sustained release over a longer period of time [233], enhanced local permeability, and efficacy at lower doses given the small size of nanosystems and their increased surface area, targeted drug, reduction in side effects, biocompatibility, biodegradability, and lack of toxicity [232,234,235].

The existing literature presents two main types of nanocarriers used for the topical treatment of psoriasis as follows: lipid-based nanocarriers and polymeric nanomaterials.

### 7.1. Lipid-Based Nanocarriers (NLBCs)

NLBCs represent promising encapsulation and drug delivery systems for the pharmaceutical industry, given that they are easy to prepare, have low production costs, and provide good bioavailability [236,237]. They have better thermal stability compared to other types of nanoparticles (NPs) and their constituents are also more biodegradable and biocompatible compared to synthetic polymeric constituents [237]. Moreover, adverse effects are diminished given the reduced toxicity potential [236,238]. NLBCs can transport both hydrophilic and lipophilic active molecules, which are gradually released at the target site and then degraded [238,239]. This category includes vesicular systems (liposomes, niosomes, transferosomes, and ethosomes), solid lipid NPs, nanostructured lipid carriers, and nanoemulsions.

#### 7.1.1. Liposomes (LIPs)

LIPs are spherical organic NPs consisting of a double membrane surrounding a core. The double membrane consists of a mixture of lipid substances (especially phospholipids and cholesterol), and the core is made up of water or buffer solutions [238,240]. This structure has the advantage of encapsulating a wide variety of substances, given that it can incorporate both polar substances which will be included in the aqueous environment, and non-polar molecules that are interspersed between the lipidic bilayer. Moreover, the enclosed aqueous core protects the incorporated substances, thus reaching target locations [240,241]. The disadvantages would be represented by a certain degree of toxicity and stability problems based on pH [242].

Their skin permeability can be correlated with the types of lipids used, the lipid–drug ratio, the surface charge, and the particle size. Moreover, the lipid content influences the elasticity, size of particles, entrapment of substance, and permeability [243]. The most commonly used phospholipid is phosphatidylcholine, which can be of natural or synthetic origin, the natural one presenting lower toxicity [244]. It is mainly derived from soy or egg sources, having distinct characteristics considering the saturation of fatty acids [243]. It is also very well tolerated by the skin, thus reducing the risk of unwanted reactions (e.g., hypersensitivity), given it is the most abundant lipid component of the cell membrane [245]. Phosphatidylcholine at 32 °C reaches a fluid state, which leads to an easier partitioning of the active substance in the deeper layers of the skin. In order to offer a certain rigidity to the structure of LIPs, cholesterol is added, the increase in its percentage determining the increase in the free energy barrier necessary for the flip–flop movement of phospholipids, the increase in the bilayer thickness, and implicitly the decrease in deformability [246]. On the other hand, cholesterol decreases the loading efficiency of hydrophilic substances, since when increasing the bilayer thickness, the size of the core decreases [247]. In addition, given the increase in rigidity, permeability becomes limited [248]. J. Li et al. observed that as the particle size decreases, the loading efficiency and in vitro permeability increase. This is explained by the decrease in the amount of cholesterol, which leads to a decrease in the rigidity and thickness of the bilayer, and implicitly to a better entrapment of the active substance [243].

Gupta et al. prepared LIPs containing capsaicin by the thin-film hydration method, using soy phosphatidylcholine and cholesterol. The obtained vesicles were spherical in shape and nanometric in size, with an entrapment efficiency of 70.98%. Since capsaicin is lipophilic, it was found to be included in the lipid layer. The LIPs were incorporated in Carbopol-934 gel and the amount of capsaicin that permeated through the skin in 24 h was 19.67 µg/cm^2^. This quantity was smaller than that provided by a gel formulation of emulsomes (capsaicin, phosphatidylcholine, and tristearine) (32.78 µg/cm^2^) [249].

Another well-known substance used in the treatment of psoriasis is psoralen. This compound, when used in conventional formulations such as tinctures and ointments, has a low permeability, requires frequent administration, and can cause adverse reactions, such as severe burning, blisters, and pigmentation, and can even pose a risk for skin cancer [250,251]. Two types of LIPs containing psoralen were prepared: one cationic (containing 3ß-[N-(N′,N′-dimethylaminoethane)-carbamoyl] cholesterol hydrochloride and cholesterol) and one anionic (containing egg lecithin, 1,1′,2,2′-tetramyristoyl cardiolipin, and cholesterol). The entrapment efficiency was 75.12% for the cationic LIPs and 60.08% for the anionic LIPs. Comparing the in vitro release profile of psoralen from LIPs and a liposomal gel (by dispersion of optimized LIPs in 15% hydroxypropyl methyl cellulose E15 gel bases), the release was slower (32.79% for the cationic formulation and 32.73% for the anionic formulation) than that of LIPs (54.14% and 62.98%) in 18 h. Implicitly, the release of psoralen was sustained over time, the skin deposition and penetration of the substance were enhanced, and some of the adverse reactions were avoided [251].

#### 7.1.2. Niosomes (NIOs)

NIOs are drug transport vesicles similar to LIPs, composed of two layers and an aqueous cavity. Unlike LIPs that contain a concentric bilayer of phospholipids, NIOs contain amphiphilic non-ionic surfactants (Brij: alkyl ethers and alkyl glyceryl ethers, polyoxyethylene 4 lauryl ether, polyoxyethylene cetyl ether, polyoxyethylene stearyl ethers; spans: sorbitan fatty acid esters; Tween: polyoxyethylene fatty acid esters; and pluronic: copolymers made up of polyethylene oxide and polypropylene oxide), and lipids (cholesterol, L-α-soya phosphatidylcholine) [241,252]. In the case of NIOs, both hydrophilic and hydrophobic substances can be incorporated, with the hydrophilic ones being found in the aqueous part or adsorbed on the bilayer surface, and the hydrophobic ones trapped inside the double layer [253]. Some of the most important advantages of using NIOs are represented by the higher skin permeation capacity, targeted, controlled, or sustained activity, better chemical stability, controlled shape, size, and composition, encapsulation of higher amounts of substances, biodegradability, biocompatibility, longer shelf life, and decreased toxicity [253,254].

Capsaicin was also formulated as NIOs, using Span 80 and cholesterol. The vesicles were spherical in shape and nanometric in size, with an entrapment efficiency of 54.30%. Moreover, after incorporation in gel, the quantity of capsaicin permeated through the skin in 24 h was smaller than in the case of LIPs [249].

Another compound formulated as NIOs is celastrol, a pentacyclic triterpene isolated from *Tripterygium wilfordii* with antioxidant, anti-inflammatory, and anti-tumor properties, with poor water solubility. For topical administration in the treatment of psoriasis, NIOs with celastrol were prepared by the film hydration method using sonication. The employed components were cholesterol, as a nonionic surfactant, and a mixture of Span 20 and Span 60 to increase the elasticity of the formed niosomes. The weight ratio of Span 20:Span 60:cholesterol was 3:1:1. The obtained particles were spherical in shape and displayed good colloidal stability, a negative zeta potential, and an entrapment efficiency of 90.42%. Comparing the results for in vitro permeation studies for NIOs vs. celastrol, a 13-fold improvement was observed in the case of NIOs due to increased solubility and skin permeation. Regarding in vivo testing using psoriasis mouse models, NIOs reduced efficiently both the white scales and erythema and the spleen/body weight ratio, as well as the levels of IL-22, IL-23p40, IL-17, and IFN-γ. Consequently, NIOs have promising potential in psoriasis therapy [255].

#### 7.1.3. Transferosomes (TRAs)

TRAs are ultra-deformable vesicles for transdermal applications, consisting of an ethanol/aqueous core, a phospholipid bilayer, and an edge activator (surfactant molecule). Depending on its hydrophilic or hydrophobic character, the active substance can be encapsulated in the core or between the lipid bilayers. Compared to LIPs, TRAs can penetrate into the deep layers of the skin after topical administration, providing higher concentrations of active substances. An osmotic gradient is created, and the substances reach the stratum corneum both intracellularly and transcellularly [245,256]. Moreover, TRAs can deliver transdermally both high molecular weight (peptides, proteins, and nucleic acids) and low molecular weight substances [257]. 

The lipid bilayer is mainly composed of soy phosphatidylcholine and egg/soy lecithin (soy lecithin is preferred if the drug is mainly hydrophobic) [258,259]. Phospholipids influence the zeta potential and the size of TRAs, as well as the encapsulation efficiency and permeability [258].

Edge activators have the role of influencing the entrapment, flexibility, and permeability of the substance. It also influences the size of TRAs, the increase in the surfactant content causing a decrease in their size. Edge activators are usually surfactants (sodium cholate, sodium deoxycholate, sodium lauryl sulfate, Tweens, Spans, Brij, Stearylamine, dodecyltrimethylammonium bromide, and cetylpyridinium chloride monohydrate) or oils (oleic acid, eucalyptus oil, and castor oil). Increasing the amount of edge activator increases the deformability of the vesicular membrane, which changes the organization of surfactant molecules in the lipid structure, creating pores in the membrane and therefore causing substance leakage. Positively charged particles have better permeability compared to neutral or negatively charged ones. Surfactants also open skin pores to improve drug permeability [260]. Therefore, the optimal ratio between synthesis components and the critical parameters of the process influence the development of TRAs [245].

Some of the most notable advantages of TRAs are their improved bioavailability, protection from metabolic degradation of the active substance, and their accepted use for substances with a narrow therapeutic window [261]. 

One example of TRAs that could be employed in the treatment of psoriasis consisted of vitamin E-loaded TRAs (soy lecithin, edge activator: sodium deoxycholate) in *Aloe vera* gel. The obtained TRAs were spherical in shape and presented a highly negative surface charge. The entrapment efficiency was 92.29% and the formula demonstrated a prolonged release of active substance and good skin compatibility [262].

#### 7.1.4. Ethosomes (ETOs)

ETOs are novel lipid-based vesicular systems that demonstrate unique improvements in substance delivery compared to other nanosystems, as they allow the transport of large amounts of active substances that can penetrate into the deep layers of the skin and/or into systemic circulation [263]. They also support the release of the substance (that can be either hydrophilic or lipophilic), which is protected from the environment [264].

ETOs mainly contain a phospholipid double layer (phosphatidylcholine, 1,2-dipalmitoyl-sn-glycero-3-phosphatidylglycerol, or 1,2-dioleoyl-3-trimethylammonium-propane chloride salt), an aqueous compartment (water or buffer solutions), and ethanol [265,266]. Ethanol is used because it improves flexibility and deformability and contributes to the increase in the fluidity of lipids from cell membranes, which determines better penetration into dermal layers [263]. ETOs’ size, entrapment efficiency, and permeability depend largely on the phospholipids:ethanol ratio [267]. 

As previously mentioned, psoralen can be formulated as LIPs, thus showing improved activity. Pierre and dos Santos Miranda Costa claim that if the size of the particles is irregular, they cannot penetrate deep into the epidermis [268]. Therefore, the study undertaken by Zhang et al. aimed to synthesize ETOs based on psoralen and compare its release, permeability, and maintenance at the skin level vs. a conventional formula (tincture). The injection method was used for preparation, and in addition to the active substance, Lipoid S 100 (phosphatidylcholine from soybean lecithin) and ethanol were included. The obtained ETOs were uniform in size, and it was found that the smaller ones had excellent deformability and implicitly penetrated the stratum corneum more easily. The formulation used in subsequent tests contained Lipoid S 100 5% *w*/*v* and ethanol 40% *v*/*v* and presented an 85.62% entrapment efficiency. In vitro skin permeation studies demonstrated an increase in transdermal delivery and flow across the skin compared to the tincture. Moreover, depending on the area of administration, the best results were obtained after application on the abdomen, compared to the chest and scapulas. Regarding the determination of skin deposition after 24 h, in the case of ETOs, the psoralen concentration was clearly higher than that of the tincture. The conclusion of the study was that improved permeability and skin deposition of psoralen delivered by ETOs may help reduce toxicity and improve long-term treatment efficacy [269].

Thymoquinone, the main component of the *Nigella sativa* essential oil, is a benzoquinone with high hydrophobicity, low water solubility, low bioavailability, and decreased chemical stability. Therefore, in order to improve its solubility and increase its therapeutic concentration at the site of action, Negi et al. prepared ETOs using the cold method with Phospholipon 90G and thymoquinone dissolved in ethanol. It was demonstrated that the use of ethanol offers, on one hand, a higher loading of the active substance, thus a higher concentration at the site of action, and on the other hand, better penetration into skin layers with prolonged release. The formula chosen for testing contained 10% ethanol, a percentage regarded as safe for topical administration, fitting at the same time into the optimal concentration for increased dermal penetration. The prepared ETOs had an entrapment efficiency of 79.52%. The optimized formula was incorporated into Carbopol 934 hydrogel (1% *w*/*w*) and tested in vivo in a mouse-tail model for antipsoriatic potential. The results demonstrated that the gel loaded with ETOs presented an increased efficacy of action compared to thymoquinone, possibly due to the large surface area of the nanoformulation found in contact with the epithelium and implicitly to the higher concentration of the substance maintained at the site of action [270].

Another study aimed at targeting the overexpressed CD44 in inflamed psoriatic skin using a curcumin formulation of propylene glycol-based ETOs covalently linked to hyaluronic acid as a vehicle. In vivo testing was conducted on an imiquimod-induced psoriasis mouse model, comparing curcumin-loaded hyaluronic acid-modified ETOs, curcumin-loaded ETOs, and a curcumin 25% propylene glycol solution. Hyaluronic acid was chosen since it is a natural ligand used to improve targeting efficiency and increase drug concentration at the site of action, thus enhancing the therapeutic effect while reducing side effects. The highest cumulative transdermal amount and skin retention of curcumin were found in the case of curcumin-loaded hyaluronic acid-modified ETOs and the lowest for the curcumin propylene glycol solution. It is presumed that hyaluronic acid forms a gel on the surface that reduces the leakage of curcumin. The active substance release was slow, which led to a higher improvement of inflammation symptoms in the case of curcumin-loaded hyaluronic acid-modified ETOs, as demonstrated by the downregulation of cytokines such as TNF-α, IL-17A, IL-17F, IL-22, and IL-1β [271]. 

#### 7.1.5. Solid Lipid Nanoparticles (SLNs) and Nanostructured Lipid Carriers (NLCs)

SLNs are colloidal drug delivery carriers that are widely used in topical applications to improve active substance accumulation and local deposits formation. In order to increase permeability, they are generally administered under occlusion [240]. They contain a mix of solid lipids stabilized by emulsifiers [272], present chemical and physical stability, site-specific targeting, low cost, and can incorporate both hydrophilic and hydrophobic molecules [273]. Thus, when formulating SLNs, lipids, emulsifiers, and active substances must be carefully considered, along with an appropriate solvent system [241]. However, given their crystalline structure, the loading capacity is low, and the dense “brick and mortar” skin model can prevent transdermal delivery of the substance. Furthermore, there is the possibility that the active substance is released during storage or that the substance is released uncontrollably in skin layers [272,274,275].

On the other hand, NLCs are designed as drug release control materials, overcoming some of the limitations of SLNs, exhibiting increased stability and efficient drug loading [238]. The drug is encapsulated in a mixture of unsaturated liquid or solid lipids with a solid core, containing preferably a non-crystalline matrix [276,277]. Even if they have similar preparation methods (cold and hot homogenization or hot emulsification ultrasonication), the oils used in the formulation contribute to reducing the expulsion of the substance during storage. They also have very good biocompatibility, improved permeation and retention, and exhibit occlusive properties [237].

Another thymoquinone formulation for the treatment of psoriasis consisted of SLNs. For the synthesis, two methods were combined, melt-emulsification and ultrasonication, with Tween 80 and poloxamer 188 being used as surfactants. The optimized formulation of SLNs had an 81.3% entrapment efficiency and a release rate of 77.93%. This formula was further included in a gel (Carbopol 934). The optimized thymoquinone SLN-loaded gel had a 93.35% substance content and a release rate of 57.55% after 24 h. Moreover, in the case of the SLN gel formulation, an initial rapid release was observed due to the presence of the drug on the surface, followed by a slow release due to either the increased viscosity of the gel or the trapping of the substance in the gel matrix, the diffusion process being thus prevented [278]. 

Cannabidiol is another natural agent that showed promise in the treatment of psoriasis. SLNs containing three types of lipids (cetyl alcohol, lauric acid, and a stearic–lauric acid mixture) were tested on an imiquimod-induced psoriasis model. The reduction in IL-17A secretion was different, with a higher anti-inflammatory effect being noticed for the mixture, while the SLNs containing lauric acid had no effect. Therefore, the selection of employed lipids in such formulations is important for topical delivery [279]. 

Psoralen was also formulated as SLNs and NLCs, using the hot homogenization and ultrasonication method. Glyceryl palmito-stearate (Precirol ATO 5) was chosen as the solid core of SLNs and Pluronic F68 and Myverol 18-04 K as surfactants. In the case of NLCs, the core consisted of Precirol ATO 5 and squalene, and two mixtures of Pluronic F68 and Myverol 18-04 K, and Tween 80 and hydrogenated soybean phosphatidylcholine, respectively, that were used as surfactants. When comparing the results obtained for NLCs with those for SLNs considering permeation and controlled release, better values were obtained for NLCs, which could be explained by the smaller size, which is equivalent to greater solubility and better occlusive effects through the formation of a film on the skin surface [280].

Another study aimed to compare two formulations incorporating capsaicin in SLNs (with Compritol 888 ATO as the lipid phase and L-α Egg phosphatidylcholine as the emulsifier) and NLCs (Compritol 888 ATO and oleic acid as the lipid phase and L-α Egg phosphatidylcholine and Pluronic F68 as the emulsifiers), prepared by the solvent diffusion method. The size of NLCs was smaller than that of SLNs. However, the entrapment efficiency and the amount of capsaicin accumulated in the skin were higher in the case of NLCs, and no skin irritation was observed. Therefore, NLCs could represent a better option when formulating capsaicin for topical delivery [281].

#### 7.1.6. Nanoemulsions (NEs)

NEs represent colloidal particulate systems that contain two immiscible liquid phases (e.g., coconut, corn or olive oil, and water) that form a single phase by the use of emulsifying agents (surfactant, co-surfactant). Different advantages of NEs are reported in the literature, such as enhanced physical stability and bioavailability of active substances, solubilization of lipophilic substances, improved thermodynamic activity, which determines a better partitioning of the substance into the skin, as well as a lack of toxicity [282,283,284].

Algahtani et al. prepared NEs with curcumin by employing the low-energy emulsification method, which were embedded in Carbopol 934 gel. For the synthesis, several types of oils, surfactants, co-surfactants, and stabilizers were assessed. The best option chosen for subsequent testing contained propylene glycol dicaprylocaprate and diethylene glycol monoethyl ether for solubilization in the oil system and for improving the loading of curcumin in the oily phase, Tween 20 as a surfactant, and polyethylene glycol (15)-hydroxy stearate as a co-surfactant. The best in vitro curcumin release was 82% for the smallest droplet size obtained. Comparing the permeation and the amount of curcumin deposited in the skin of NE curcumin-loaded gel vs. curcumin gel, the results demonstrated a 4.87-fold increase in permeation and a higher quantity of curcumin in the first case, which proves that such a formulation can improve the long-term treatment of psoriasis [285]. 

### 7.2. Polymeric Nanomaterials

Polymeric nanomaterials are colloidal structures made of nanosized molecules of natural or synthetic polymers with particles ranging between 10 and 1000 nm. They are widely used in the medical and pharmaceutical field as efficient drug-delivery systems as they confer high skin penetration, thus providing controlled release at targeted areas [185]. Polymeric nanomaterials represent an efficient tool for topical drug delivery systems in psoriasis when drug penetration is severely affected by the abnormal thickening of the stratum corneum due to the hyperproliferation of keratinocytes [286]. Such nanomaterials are characterized by well-controllable sizes, surface polarity, and high reactivity for chemical modifications, therefore allowing functionalization with active compounds and adaptations to the intended target [287]. Moreover, the long circulation time in the organism, biocompatibility, biodegradability, nonimmunogenicity, as well as the availability of various synthesis methods make polymeric nanomaterials excellent candidates as nanocarriers for drug delivery [286].

As drug delivery systems, such materials may retain drug molecules by different processes, such as encapsulation, dispersion, adsorption, or conjugation with a suitable polymer matrix. Several techniques are used to obtain drug-loaded polymeric nanostructures such as polymerization and top-down or bottom-up methods [288]. Polymeric nanosystems may be classified as non-self-assembled and self-assembled. Polymeric NPs (nanospheres, nanocapsules), dendrimers, and polymeric micelles are non-self-assembled polymeric nanomaterials. Nanospheres consist of a matrix in which the active substance is dispersed, while nanocapsules have a core-shell structure with the active substance encapsulated in the core [289]. The drug may also be adsorbed or conjugated to the polymeric nanoparticle’s surface. Dendrimers have a three-dimensional structure, conferred by the use of highly branched polymers in their synthesis. Antipsoriatic agents are retained through covalent or electrostatic bonds formed with the dendrimer core or surface [286]. Polymeric nanomicelles are synthesized through the polymerization of amphiphilic block copolymers that self-assemble into nanoscale micelles [287]. Nanogels are self-assembled polymeric nanomaterials formulated as a cross-linked polymeric network with a high capacity to load antipsoriatic agents [289]. 

As drug-delivery systems, polymeric nanomaterials present an important advantage given by their capacity to load both hydrophilic and hydrophobic substances. This outcome may be achieved by selecting the proper polymers and synthesis methods. Polymers show a variety of specific characteristics and may be natural (chitosan, silk fibroin, albumin, gelatin, and dextran) or synthetic such as polylactic co-glycolic acid (PLGA), polyethylene glycol (PEG), poloxamers, polyplexes, polylactic acid-polycaprolactone-polyglycolic acid (PLA-PCL-PGA), polyamidoamine/polypropyleneimine (PAMAM/PPI), polyhydroxylpropylmethacrylamide (PHPMA), and polyalkylcyanoacrylates (PACA) [286]. 

#### 7.2.1. Polymeric Nanoparticles

Polymeric NPs are particulate polymeric nanosystems that are carriers of various active compounds. This category mainly includes nanospheres and nanocapsules, both having dimensions that vary between 1 and 1000 nm [290]. The active compounds are either entrapped in the polymeric core or adsorbed on the particle surface. Their main advantage consists of the ability to control drug release at the specific target, therefore having reduced toxicity. Moreover, they show high stability and increased bioavailability for both hydrophilic and lipophilic drugs. Substances entrapped in nanospheres and nanocapsules reach the desired target following diffusion or erosion of the polymer matrix [185]. In topical delivery, polymeric NPs show a significant degree of accumulation in the stratum corneum of the skin. In contrast, emulsion nanosystems concentrate in the dermis. When compared to lipidic nanomaterials, polymeric nanosystems form deposits in the skin, which indicates the latter as being more suitable for topical treatment [291]. Furthermore, charged polymeric NPs have a superior retention time in tissues associated with the inflammation process compared to healthy skin [292]. As psoriasis is a pathology that involves inflammation of the skin’s outermost layer (stratum corneum), the use of drug-loaded polymeric NPs for the local treatment of psoriasis represents an efficient approach. 

Curcumin is one of the most studied natural active compounds for psoriasis treatment, with several nano-formulations for curcumin encapsulation being proposed until present. Curcumin (1,7-bis(4-hydroxy-3-methoxyphenyl)-1,6-heptadiene3,5-dione) is the main active compound extracted from *Curcuma longa* rhizomes and has various biological activities, such as antioxidant, anti-inflammatory and antiproliferative functions. As psoriasis pathogenic pathways mainly include T-cell-mediated inflammation and keratinocyte proliferation, curcumin holds promise as a suitable candidate [293]. Bilia et al. synthesized polymeric curcumin NPs with poly(vinylpyrrolidinone)-30 (PVP30) with the intention to enhance solubility, stability, and permeability of the active compound [205]. To test their effectiveness, these curcumin-loaded NPs were orally administered to patients with moderate-to-severe psoriasis (PASI > 10) in a placebo-controlled, double-blind, randomized clinical trial. Results showed that nanocurcumin treatment had a significant impact on the reduction in PASI score and, moreover, represented a benefic adjuvant therapy for acitretin, by maintaining cholesterol serum levels unchanged. Other curcumin particles were designed by Mao et al. using the amphiphilic polymer RRR-α-tocopheryl succinate-grafted-polylysine conjugate, in order to be suitable for topical delivery [294]. The synthesized NPs showed an encapsulation efficiency of 78.45% and were further incorporated in a silk fibroin as a hydrogel matrix to increase the topical release of curcumin. The novel nanomaterial inhibited the expression of inflammatory cytokines (TNF-α, IL-1β) and showed effective anti-keratinization activity in the in vivo studies on an imiquimod-induced psoriatic-like plaque mice model. Similarly, curcumin-loaded poly (lactic-co-glycolic acid) (PLGA) nanoparticles were designed by Sun et al. and their antipsoriatic efficiency for topical treatment was evaluated on an imiquimod-induced psoriatic-like plaque mice model [295]. The authors compared two different sizes of nanocurcumin (50 vs. 150 nm) and concluded that the smaller ones have improved antipsoriatic activity by analyzing the results of the morphological evaluation, biomarkers, and protein levels.

Another study investigated the capacity of chitosan-based Tween 80-coated NPs to deliver gallic acid and rutin targeting keratinocyte hyperproliferation, tissue inflammation, oxidative species production, and microbial infection associated with psoriasis [296]. Gallic acid (3,4,5-trihydroxybenzoic acid) and rutin (3′,4′,5,7-tetrahydroxy-flavone-3-rutinoside) are natural phenolic compounds that have multiple biological activities, such as anti-inflammatory, antimicrobial, anticancer and antioxidant functions [297,298]. The in vitro drug release study showed a sustained release pattern for gallic acid and rutin, as 76% of the total content in active compounds was delivered in 16 h. As evaluated on the HaCaT cell line, the anti-keratinocytes hyperproliferation activity was defined by an IC_50_ value of 115.7 μg/mL [296].

#### 7.2.2. Dendrimers

Dendrimers are branch-like structures of nanoscale dimensions. They have spherical shapes, sizes of less than 15 nm, and a central core encircled by peripheral functional groups that may chemically bind to other molecules [238,299]. The interactions between dendrimers and biological substrates, such as those with lipid bilayers, may be modulated by polymer size, charge, and chemical composition. Through encapsulation of both hydrophilic and hydrophobic antipsoriatic drugs in dendrimers, the research aims to improve skin penetration, as well as biological effects [300]. Dendrimers show notable enhancement in drug permeation and are reported to possess anti-inflammatory properties while also reducing skin irritation [287].

Yu et al. designed erianin-loaded photo-responsive dendrimer mesoporous silica NPs for the local treatment of psoriasis [301]. Erianin is a natural bibenzyl derivative (2-methoxy-5-[2-(3,4,5-trimethoxy-phenyl)-ethyl]-phenol) extracted from *Dendrobium chrysotoxum.* It is reported to possess anti-tumor effects, along with antibacterial, antiviral, anti-inflammatory, and antioxidant activities [302]. Erianin also shows effective antipsoriatic activity by the inhibition of proliferation and apoptosis inducement in the spontaneously immortalized human keratinocyte cell line (HaCat). However, it has some important drawbacks that limit its topical administration, such as poor water solubility and low skin penetration. The light-triggered dendritic mesoporous silica loaded with erianin showed an enhanced HaCat cell-inhibiting efficacy, with cell viability of only 10% (500 mg/mL). Moreover, microscopy studies indicated efficient cellular uptake. Therefore, this novel dendrimer-type nanomaterial proved to be a suitable carrier for erianin, improving its bioavailability and showing sustained release effects [301]. Another in vivo study that investigated the dermal penetration of dendritic core–multishell nanocarriers (PG_10,000_(-NH_2_)_0_._7_(C_18_-mPEG_6_)_1_._0_) in a murine skin model of psoriasis showed that topical administration leads to the accumulation of these nanomaterials in the stratum corneum, with no permeation to deeper viable skin layers. Moreover, these dendritic structures were completely biocompatible with both normal and psoriatic mouse skin, while also having the ability to enhance nile red (the model for lipophilic compounds) penetration into the viable epidermidis [303].

#### 7.2.3. Polymeric Micelles

Polymeric micelles are synthesized from amphiphilic block copolymers that self-assemble to produce a unique configuration. These colloidal systems have a hydrophobic core and hydrophilic shell, with sizes that range between 5 and 200 nm [304]. Drug-loaded polymeric micelles are characterized by a higher stability in physiological solutions, enhanced permeability, controlled release with target specificity, and longer circulation [305]. The particular configuration of polymeric micelles confers increased entrapment, enhanced solubility, and the bioavailability of antipsoriatic molecules [185]. The surfactant characteristics of these micelles cause temporary damage in cell arrangement, which facilitates the absorption of poorly soluble drug molecules (para-cellular absorption) [306].

Khurana et al. tested the antipsoriatic activity (through in vitro, ex vivo, and in vivo studies) of polymeric micelles synthesized using a mixture of Polaxomer F127 and P123, loaded with resveratrol and further embedded in a carbomer-based hydrogel [307]. Resveratrol (3,5,4′-trihydroxystilbene) is a natural polyphenol with a stilbene structure, characterized by low water solubility. Resveratrol has several important biological activities such as antioxidant, anti-tumor, and anti-inflammatory properties, that could prove useful in psoriasis treatment [308]. As polymeric micelles have enhanced skin permeation properties, skin compatibility and high stability, they were selected as suitable drug-carriers for resveratrol, intended for topical psoriasis treatment. In vivo tests revealed a better skin permeation for the novel polymeric micelles-type nanomaterial and enhanced deposition of resveratrol in deeper skin layers, evaluated in an imiquimod-induced psoriatic-like plaque model. Moreover, a DPPH assay proved that the antioxidant activity of resveratrol was not affected by encapsulation [307]. 

Another novel carrier system represented by a combination between nanomicelles and nanoemulsions containing aceclofenac and capsaicin entrapped in carbopol gel was compared to a marketed formulation (Aceproxyvon) considering the activity in an imiquimod-induced psoriatic-like plaque model developed on mice. The research proved that the novel nanotechnological system was superior to the marketed product. An enhancement in skin permeation of aceclofenac and capsaicin, translocation of the active substances into the deeper layers of the skin, improvement of skin contact time, as well as a beneficial occlusive effect could be observed [306].

#### 7.2.4. Nanogels

Nanogels are polymeric crosslinked networks with a hydrophilic nature, with pores that have a high capacity to encapsulate bioactive molecules. Specific properties may be achieved by the proper selection of polymers and crosslinkers used during synthesis [286,287]. The antipsoriatic efficacy of a nanohydrogel synthesized from micellar choline-calix[4]arene amphiphile and curcumin was evaluated on an imiquimod-induced psoriasis model by Filippone et al. [309]. Treatment with curcumin nanohydrogel restored the expression of ZO-1 and occludin tight junctions, re-establishing the normal selective permeability of the skin. Benefic activities were also observed in the immune system, with the number of mast cells and their degranulation being significantly reduced. Moreover, the novel nanogel reduced the expression of inflammatory markers involved in psoriasis progression (TNF-α, IL-1β, and iNOS). 

Another nanogel was prepared using chitin through a regeneration process with the addition of excess methanol. The obtained nanogel was further loaded with aloe-emodin with an entrapment efficiency of around 89% [310]. Aloe-emodin (1,8-dihydroxi-3-(hydroxymethyl)-9,10-anthraquinone) is an anthraquinone derivative extracted from *Aloe vera* latex that possess significant anti-inflammatory, antiviral, antiproliferative, analgesic, and wound healing properties, that could prove beneficial for treating psoriasis. The lipophilicity of aloe-emodin increases the hydrophobic nature of the synthesized nanogel, which improves permeation via transdermal penetration. In ex vivo skin permeation studies, aloe-emodin chitin nanogel showed two-fold increased permeation compared to the control (aloe-emodin solution). Aloe-emodin tended to concentrate in the deeper layers of the skin, the epidermis and dermis, reaching concentrations of 92.73 and 126.06 μg/cm^2^. Moreover, results from the in vitro hemolysis assay and in vivo skin irritation study confirmed biocompatibility. The in vivo antipsoriatic activity of nanogels on Perry’s mouse tail model implied orthokeratosis enhancement, epidermal thickness reduction, and regular elongation of rete ridges.

#### 7.2.5. Nanosponges

Nanosponges are unique nanomaterials composed of hydrogel particles with a large surface area. They have a three-dimensional structure made from long-chain polyesters that are connected by crosslinkers having an amphiphilic nature; consequently, they present a hydrophobic chamber and external hydrophilic structures. Nanosponges may encapsulate both hydrophilic and lipophilic active compounds for which they modulate solubility and extend the release period. Since nanosponges have a permeable nature, active substances are delivered as the medium permeates inside the nanomaterial, providing a sustained release up to 24 h [311,312]. Iriventi et al. prepared a nanosponge formula consisting of beta-cyclodextrin as a polymer and dimethyl carbonate as a crosslinker, in which curcumin and caffeine were loaded, for the local treatment of psoriasis [313]. In the mouse model of imiquimod-induced psoriasis, the curcumin–caffeine nanosponge showed sustained release for 12 h. Moreover, the nanosponge formula pointed to a quicker installation of antipsoriatic activity compared to the treatment with curcumin alone (10 vs. 20 days). 

#### 7.2.6. Nanofibers

Nanofibers are defined as fibers with diameters in the nanometer range (1 nm–1 μm), synthesized from both natural and/or synthetic polymers. Nanofibers present unique properties, such as a large surface area-to-volume ratio, high porosity, mechanical strength, and easy surface functionalization with other molecules. These nanomaterials are widely used as drug-delivery systems, as their formulation and design may be easily optimized for a specific active compound release. As drug-delivery systems, nanofibers are impressively versatile, as they may confer both immediate or modified active compound release [314]. 

Capsaicin was encapsulated in nanofibers obtained through the electrospinning of poly(methyl vinyl ether-alt-maleic ethyl monoester), with an encapsulation efficiency of approximately 100%. The drug carrier showed superior stability, as capsaicin content showed no significant loss 15 days after encapsulation. Encapsulation of capsaicin in nanofibers maintained and even enhanced the capacity of the active compound to activate the transient receptor potential cation channel 1, which is associated with the formation of psoriatic lesions. Moreover, it was demonstrated that the proposed nanofiber formula is a suitable choice for the preparation of skin adhesive dressings and that the release of capsaicin takes place along with the disintegration of the nanostructure [315].

## 8. Conclusions

The numerous biological activities exhibited by natural compounds, including their anti-inflammatory, antioxidant, and immunomodulatory properties prove their potential therapeutic value for psoriasis. While conventional therapeutic approaches and biological therapies remain the mainstay of psoriasis treatment, their limitations in terms of contraindications and side effects emphasize the need for alternative therapeutic agents. Natural compounds, such as polyphenols (e.g., resveratrol, curcumin, and quercetin), alkaloids (e.g., capsaicin, colchicine, and indirubin), coumarins (psoralen and methoxypsoralen), and terpenoids (e.g., celastrol, limonene) hold promise as adjunctive or alternative treatments for psoriasis, given their complex and various mechanisms. Considering the numerous advantages it offers, such as protection of the active substance, enhanced local permeability, biocompatibility, and biodegradability, nanotechnology can be successfully used for the implementation of such novel therapeutic agents. Nevertheless, further research and clinical studies focusing on fully understanding the mechanisms of action of these natural compounds are essential for expanding current treatment options and improving psoriasis management.

## Figures and Tables

**Table 1:** Medicinal plants with potential use in the treatment of psoriasis.

Species (Family)	Part(s) of Plant Used as Such or for Extraction	Application	Natural Constituents with Potential Implications	Reference
* Aleurites moluccana * *(Euphorbiaceae)*	Seeds (Oil)	Topical	Polyunsaturated fatty acids	[45,46,47]
* Aloe vera * *(Asphodelaceae)*	Leaves (Gel)	Topical	Polysaccharides, glycoproteins, anthraquinones, and salicylic acid	[48,49,50]
* Ammi majus * *(Apiaceae)*	Fruits	Topical, oral	8-methoxypsoralen	[47,51,52]
* Andira araroba * *(Fabaceae)*	Bark	Topical	Anthraquinones (chrysarobin)	[47,48,51]
* Angelica sinensis * *(Apiaceae)*	Roots	Topical	Furanocoumarins (psoralen), ferulic acid	[53]
* Anthemis cotula * *(Asteraceae)*	Flowers	Topical	Polyphenols	[54]
* Arctium lappa * *(Asteraceae)*	Leaves	Topical	Lignans, polyphenols	[49,55]
*Artemisia capillaris * *(Asteraceae)*	Whole plant	Topical	Flavonoids, coumarins, and chlorogenic acids	[56,57]
* Avena sativa * *(Poaceae)*	Seeds	Topical	Polyphenols, proteins, and lipids	[49,58]
* Azadirachta indica * *(Meliaceae)*	Leaves	Oral	Nimbidin, quercetin, and β-sitosterol	[59,60]
* Baphicacanthus cusia * *sin. Strobilanthes cusia* *(Acanthaceae)*	Leaves, stems	Topical	Indirubin	[47,51,61]
* Calendula officinalis * *(Asteraceae)*	Flowers	Topical	Flavonoids, carotenoids, and volatile oil	[62,63]
* Camptotheca acuminata * *(Nyssaceae)*	Bark, stem	Topical	Alkaloids (camptothecin)	[47,64,65]
* Cannabis sativa * *(Cannabinaceae)*	Hemp seed oil	Topical	Unsaturated fatty acids, phytocannabinoids	[66,67,68]
* Capsicum frutescens * *(Solanaceae)*	Fruits (cream)	Topical	Capsaicin	[47,69,70]
* Catharanthus roseus * *(Apocynaceae)*	Leaves, stems	Topical	Alkaloids	[71,72]
* Celastrus orbiculatus * *(Celastraceae)*	Celastrol (triterpene)	Topical	Triterpenoids, flavonoids, and sesquiterpenoids	[73,74]
* Centella asiatica * (*Apiaceae*)	Aerial parts	Topical	Triterpenoid glycosides, phenolic compounds	[47,75]
* Copaifera langsdorffii * (*Fabaceae*)	Oleoresin	Topical	Diterpenes, sesquiterpenes	[64,76]
* Coptis chinensis * *(Ranunculaceae)*	Rhizomes	Topical	Protoberberine alkaloids	[73,77,78]
* Curcuma longa * *(Zinzigiberaceae)*	Rhizomes	Topical	Polyphenols (curcumin)	[47,79]
* Echinacea angustifolia, * *E. purpurea* *(Asteraceae)*	Roots, aerial parts (juice)	Oral	Alkylamides	[49,66,80]
* Humulus lupulus * *(Cannabinaceae)*	Flowers	Topical	α- and β-Bitter acids, volatile oil	[81]
*Hypericum perforatum * *(Hypericaceae)*	Flowers, aerial parts	Topical	Hypericin, hyperforin, and flavonoids	[47,82]
* Juniperus communis * *(Cupressaceae)*	Oil, fruits	Topical	Terpenoids, flavonoids	[83,84]
* Mahonia aquifolium * *(Berberidaceae)*	Fruits	Topical	Alkaloids (berberine)	[51,73,85]
* Malva sylvestris * *(Malvaceae)*	Leaves	Topical	Anthocyanidins	[86]
* Momordica charantia * *(Cucurbitaceae)*	Fruits (juice)	Topical	Glycosides, saponins, alkaloids, and polyphenols	[47,87,88]
* Nigella sativa * *(Ranunculaceae)*	Seeds	Topical	Flavonoids	[89,90]
* Oldenlandia diffusa * *(Rubiaceae)*	Whole plant	Oral	Terpenoids (oleanolic and ursolic acids)	[91,92]
* Persea americana, * *P. gratissima* *(Lauraceae)* *(combined with vitamin B12)*	Fruit (oil)	Topical	Sterols, tocopherols, squalene, lipidic furans, vitamin E, lecithin, and fatty acids	[47,51]
* Phellodendron amurense * *(Rutaceae)*	Root bark	Topical	Protoberberine alkaloids	[77,78]
*Psoralea corylifolia * *(Fabaceae)*	Seeds	Topical	Coumarins (8-methoxypsoralen, isopsoralen), and terpenes	[93]
* Rehmannia glutinosa * *(Orobanchaceae)*	Root	Oral	Iridoids (catalpol)	[94]
* Rubia cordifolia * *(Rubiaceae)*	Root	Topical	Anthraquinone and derivatives (alizarin, rubiadin, and mollugin)	[62,73,95]
* Salix alba * *(Salicaceae)*	Bark	Topical	Salicylic acid, salicin	[51]
* Salvia miltiorrhiza * *(Lamiaceae)*	Root	Topical	Salvianolic acid A, salvianolic acid B	[96,97]
* Saraca asoca * *(Fabaceae)*	Flower	Topical	Flavonoids	[66]
* Scutellaria baicalensis * *(Lamiaceae)*	Roots	Topical	Flavonoids (baicalin)	[77,98]
* Senna tora * *(Fabaceae)*	Seeds, leaves	Topical	Flavonoids, aloe-emodin	[62,99,100]
* Silybum marianum * *(Asteraceae)*	Fruits, seeds, leaves	Topical	Flavonolignans (silymarin), taxifolin, vitamin E, linoleic, and α-linolenic acids	[49,53,101]
* Smilax sp. * *(Smilacaceae)*	Rhizome	Topical	Saponins, flavonoids	[102,103]
* Trigonella arabica * *(Fabaceae)*	Seeds	Topical	Phytosterols	[54]
* Tripterygium wilfordii * *(Celastraceae)*	Root	Topical, oral	Terpenes (triptolide, celastrol)	[104]
* Wrightia tinctoria * *(Apocynaceae)*	Bark, leaves	Topical, oral	Sterols	[62,105]

**Table 2:** Alkaloids with potential implications in the treatment of psoriasis.

Compound	Mechanism of Action	Studied Model	Reference
*Berberine*	suppresses the JAK-STAT signaling pathway in keratinocytes	Mouse model	[106]
	downregulates cell division control protein 6 (CDC6) protein induces apoptosis and oxidative DNA damage	Skin biopsies of patients/healthy individuals HaCaT cells	[107]
*Capsaicin*	obstructs the activation of the IL-23/IL-17 pathway	Mouse model	[108]
	depletes substance P from sensory neurons in the skin	Patients	[109]
*Colchicine*	suppresses polymorphonuclear leukocytes and presents antimitotic activity	Patients	[110]
*Cyclopamine*	promotes epidermal cell differentiation normalizes EGFR expression	Patients	[111]
*Indigodoles*	presents anti-IL-17A properties	Th17 cell model	[112]
*Indirubin*	inhibits inflammatory responses mediated by IL-17 A-producing γδ T cells	Mouse model	[113]
*Khasianine*	reduces TNF-α levels in lesions suppresses NF-κB p65 activation and the expression of IL-17A and IL-33	Mouse model	[114]
*Narciclasine*	regulates lipid metabolism-related genes, particularly the phospholipase A2 family increases anti-inflammatory lipid molecules	Mouse model HaCaT cells	[115]
*Noscapine*	inhibits the generation of pro-inflammatory mediators via TNF-α/IFN-γ activation	Mouse model	[116]
*Piperine*	inhibits the phosphorylation of STAT3	Mouse model HaCaT cells	[117]
*Tryptanthrin*	suppresses inflammation and oxidative stress via the NF-κB/MAPK/Nrf2 pathways	Mouse model HaCaT cells	[118]

**Table 3:** Anthraquinones with potential mechanisms in the treatment of psoriasis.

Compound	Mechanism of Action	Studied Model	Reference
*Aloe-emodin*	diminishes IL-6 and IL-8 overexpression	Mouse model HaCaT cells	[119]
*Chrysophanol*	diminishes IL-6, IL-8, and IL-24 overexpression reduces TNF-induced cytokine production relieves local and systemic inflammation downregulates mitogen-activated protein kina ses (MAPKs) and NF-κB	Mouse model HaCaT cells	[119]
*Emodin*	diminishes IL-6 and IL-8 overexpression inhibits the ERK, STAT3, and NF-κB signaling pathways	Mouse model HaCaT cells	[119,121]
*Rhein*	diminishes IL-6, IL-8, and IL-24 overexpression reduces TNF-induced cytokine production downregulates MAPKs and NF-κB	Mouse model HaCaT cells	[119,121]

**Table 4:** Flavonoids with potential implications in the treatment of psoriasis.

Compound	Mechanism of Action	Studied Model	Reference
*Amentoflavone*	suppresses NF-κB-mediated inflammation influences several pro-inflammatory cytokines, such as TNF-α, IL-17A, IL-22, and IL-23	HaCaT cells Mouse model	[134]
*Apigenin*	inhibits the IL-23/STAT3/IL17A signaling axis inactivates NF-κB inhibits TNF-α, IL-6, and IL-1β expression	HaCaT cells Mouse model	[135]
*Astilbin*	prevents cell proliferation decreases ROS generation enhances Nrf2 activation under pro-inflammatory cytokine stress downregulates the expression of VEGF by Nrf2 activation	HaCaT cells	[136,137]
	diminishes Th17 cell differentiation and suppresses IL-17 secretion prevents T cell-mediated inflammatory infiltration restores the abnormal proliferation and differentiation of keratinocytes	Mouse model	[132]
	inhibits R837-induced maturation and activation of bone marrow-derived dendritic cells decreases the expression of pro-inflammatory cytokines	Mouse model	[138]
	inhibits p38 MAPK inhibits IL-6 and IL-22	Guinea pig model	[139]
*Baicalin*	inhibits IL-17A, IL-22, and IL-23 production	Mouse model	[98]
*Chrysin*	regulates the MAPK, JAK-STAT, and IKK/NF-κB signaling pathways	Mouse model Human primary keratinocytes	[140]
*Delphinidin*	modulates the turnover of caspase-14 and filaggrin	Reconstructed human psoriatic skin model	[141]
*Fisetin*	downregulates components of the PI3K/AkT/mTOR signaling pathway	Human skin model	[142]
*Genistein*	downregulates the expression of inflammatory cytokines, such as IL-1β, IL-6, IL-17, and TNF-α suppresses STAT3 phosphorylation	HaCaT cells Mouse model	[143]
*Glabridin*	decreases the mRNA expression of p65, iNOS, IL-1β, IL-6, IL-17A, IL-22, and IL-23	HaCaT cells Mouse models	[144]
*Hesperidin*	reduces the levels of inflammatory cytokines such as TNF-α, IL-6, and IL-1β regulates the levels of adipokines influences aerobic respiration in keratinocytes inhibits the phosphorylation of IRS-1 Ser312 and dephosphorylation of Tyr612 in keratinocytes suppresses high expression of ERK1/2	HaCaT cells Mouse models	[145,146]
*Isoliquiritigenin*	downregulates IL-6 and IL-8 through NF-κB suppression	HaCaT cells Mouse model	[147]
*Luteolin*	downregulates IL-1β, IL-6, TNF-α, IL-17A and IL-23 expression inhibits the cutaneous infiltration of macrophages, neutrophils, and T cells	RAW264.7 cells Mouse model	[148]
	inhibits the production of IL-6, IL-8, and the vascular endothelial growth factor (VEGF) lowers NF-κB activation	HaCaT cells Adult normal human epidermal keratinocytes Human subjects	[149]
*Naringin* *(with sericin)*	lowers the expression of mRNA and the production of pro-inflammatory cytokines	Human peripheral blood mononuclear cells	[150]
*Quercetin*	inhibits the NF-κB pathway improves antioxidant and anti-inflammatory status	Mouse model	[151]
*Proanthocyanidins*	modulate Th17 and Treg cells decrease the expression levels of inflammatory cytokines and STAT3	Mouse model	[152,153]
*Taxifolin*	regulates helper T cell differentiation by inhibiting transcript factors, such as T-bet, GATA-3, and RORγt inhibits the Notch1 and Jak2/Stat3 pathways	Mouse model	[154]
	inhibits mRNA expression levels of pro-inflammatory cytokines such as IL-1α, IL-1β, IL-6, and chemokines	HaCaT cells	[155]

**Table 5:** Polyphenolic acids with potential implications in psoriasis treatment.

Compound	Mechanism of Action	Studied Model	Reference
* Caffeic acid *	reduces the mRNA and protein levels of TNF-α, IL-6, and IL-1β	Human keratinocytes Mouse model	[160]
* Chlorogenic acid *	suppress the chemotactic activity of polymorphonuclear leukocyte	Guinea pig model Mouse model	[161]
* Ferulic acid *	prevents IL-17A from binding to IL-17RA, which normally activates NF-κB, therefore reducing inflammation inhibits the infiltration and the cytokine secretion of Th17 cell, dendritic cell, and granulocyte subsets	Mouse model	[162]
* Gallic acid *	inhibits the expression of keratin 16 and keratin 17 (markers of psoriasis) through Nrf2	Mouse model	[163]
	reduces the frequency of IL-17-producing cells	Patients	[164]
* Lithospermic acid *	restores skin barrier function inhibits the IL-17/IL-23 axis	Mouse model	[165]
* Rosmarinic acid *	inhibits the IL-23/Th17 axis by downregulation of the Jak2/Stat3 signal pathway	HaCaT cell line Mouse model	[166]
* Salicylic acid *	presents keratolytic activity	Patients	[40,167]

**Table 6:** Terpenoids with potential implications in the treatment of psoriasis.

Compound	Mechanism of Action	Studied Model	Reference
*α-Bisabolol*	inhibits pro-inflammatory cytokines (TNF-α and IL-6)	Peritoneal macrophage cells, TPA-induced mouse ear inflammation	[53,176]
* β-Caryophyllene *	enhances re-epithelialization	Standard murine model for wound healing	[177]
*Chamazulene*	presents anti-inflammatory activity by the inhibition of lipoxygenase and, implicitly, of leukotriene B4 formation	Rat peritoneal neutrophilic granulocytes	[178,179]
*Celastrol*	binds directly to IL-17A, thus inhibiting its signaling decreases pro-inflammatory cytokine secretion in keratinocytes	HaCaT cell culture Mouse model	[180]
*Centelloids*	reduces mRNA levels of IL-23 and IL-17 reduces keratinocyte proliferation	Mouse model	[40]
* Geraniol *	inhibits keratinocyte proliferation presents antioxidant properties	UV-induced mouse model for psoriasis	[181]
*Limonene*	controls cytokines release reduces skin inflammation	Patients aged 13–73 years, males and females	[182]
* Linalool *	reduces the expression of NF-κB, CCR6, and IL-17 restores epidermal hyperplasia and parakeratosis	Mouse model	[183]
* α-Pinene * *β-Pinene*	antioxidative properties antimicrobial properties	-	[184,185]
* Terpinen-4-ol *	reduces TNF, IL-1, IL-8, and PGE2 production impacts vasodilation and plasma extravasation	Histamine-induced weal and flare in volunteers aged 23–54 years	[186]
* Ursolic acid *	limits NF-B activation by inhibiting IKK suppresses IL-17 production	HaCaT cell culture Mouse model	[187,188]
